# A multicopy sRNA of *Listeria monocytogenes* regulates expression of the virulence adhesin LapB

**DOI:** 10.1093/nar/gku630

**Published:** 2014-07-17

**Authors:** Susanne Sievers, Eva Maria Sternkopf Lillebæk, Kirstine Jacobsen, Anja Lund, Maria Storm Mollerup, Pia Kiil Nielsen, Birgitte Haahr Kallipolitis

**Affiliations:** Department of Biochemistry and Molecular Biology, University of Southern Denmark, Odense, Denmark

## Abstract

The multicopy sRNA LhrC of the intracellular pathogen *Listeria monocytogenes* has been shown to be induced under infection-relevant conditions, but its physiological role and mechanism of action is not understood. In an attempt to pinpoint the exact terms of LhrC expression, cell envelope stress could be defined as a specific inducer of LhrC. In this process, the two-component system LisRK was shown to be indispensable for expression of all five copies of LhrC. *lapB* mRNA, encoding a cell wall associated protein that was recently identified as an important virulence factor, was disclosed to be directly bound by LhrC leading to an impediment of its translation. Although LhrC binds to Hfq, it does not require the RNA chaperone for stability or *lapB* mRNA interaction. The mechanism of LhrC-*lapB* mRNA binding was shown to involve three redundant CU-rich sites and a structural rearrangement in the sRNA. This study represents an extensive depiction of a so far uncharacterized multicopy sRNA and reveals interesting new aspects concerning its regulation, virulence association and mechanism of target binding.

## INTRODUCTION

Throughout the last decade it has been recognized that small non-coding RNAs in bacteria are not rare and singular cases, but rather play an important and extensive regulatory role (1,2). These small RNAs (sRNAs) are often involved in the adaptation to changing environmental conditions, and several studies even proved their importance for the virulence of pathogenic bacteria (3–5). A non-coding sRNA can be encoded antisense, i.e. on the opposite DNA strand of its perfect complementary mRNA target, or it may be transcribed from a distant location (trans-encoded sRNAs) and then often regulates more than one target. It can be attributed mainly to novel high-throughput technologies (high-resolution genome tiling arrays and deep sequencing), and improved bioinformatics tools, that virtually catalogs of bacterial sRNAs (RNomes) have been published throughout the last years (5–8). However, the actual challenge starts only now. What are the functions of all these newly identified sRNAs? How exactly do they act on a molecular basis? Are they important for the pathogenicity of a bacterium, and if so, could this be exploited to develop new antimicrobial strategies?

The first sRNAs of the Gram-positive pathogen *Listeria monocytogenes* were discovered in 2006 (9) and ever since comprehensive studies in the bacterium uncovered not only an enormous number of new non-coding RNAs, but also revealed specific details on their expression under infection-relevant conditions (6,10–12). Because listeriosis represents a severe health danger especially for immunocompromised people and pregnant women and their offspring (13), research on how the pathogen regulates virulence is of high public interest. New knowledge on sRNAs that are likely to be involved in pathogenesis could be a starting point of a deeper understanding in this respect.

While in Gram-negative bacteria the RNA chaperone Hfq contributes to virtually every interaction of a trans-encoded sRNA to its mRNA target (3,14), the situation is different in Gram-positive species. Several detailed studies on sRNAs in the model organism *Bacillus subtilis* revealed dispensability of Hfq for mRNA interaction, although the RNAs were mostly capable of Hfq binding (15–18), as shown for numerous other sRNAs in this organism (19). Instead, alternative proteins of *B. subtilis* are assumed to act as RNA chaperones and to facilitate sRNA-mRNA interaction in certain cases (16,20). The function and significance of Hfq for sRNA-mRNA interactions in other Gram-positive bacteria had only rarely been shown (21), while other reports are contradictory as for *Staphylococcus aureus* (22,23). Some, like *Streptococci* and *Lactobacilli*, do not even possess an Hfq homolog (14). However, the significance of the protein in *L. monocytogenes* during specific stress conditions and for virulence has been demonstrated (24), and as a paradigm the small listerial sRNA LhrA (for *Listeria* Hfq-binding RNA A) was proven to depend on Hfq in terms of stability (9) and binding to its mRNA targets (25,26). In addition to LhrA, the sRNAs LhrB and LhrC were initially identified via co-immunoprecipitation with Hfq (9). LhrC is conserved among all *Listeria* species and present in five almost identical copies that vary from 111 to 114 nt in length (Supplementary Figure S1). A putative role of the LhrC sRNAs during listerial infections can be anticipated from later studies where they were reported to be highly expressed in blood (6) and during intracellular growth in macrophages (11).

In this study we provide evidence that the LhrC sRNAs are highly induced in response to cell envelope stress and find that expression of all five *lhrC* copies strictly depends on the two-component system (TCS) LisRK. Using bioinformatics tools, the *lapB* gene, encoding a cell-wall anchored virulence adhesin, was predicted as a target for all five LhrCs. Analyses of its regulation show that expression of *lapB* is targeted by the sRNAs at the post-transcriptional level. Interestingly, interaction of LhrC to *lapB* mRNA does not follow the predicted binding scheme involving two complementary sequences in sRNA and mRNA. Apparently, the LhrC molecule contains three redundant CU-rich motifs, residing in a stem-loop structure, a single-stranded region and the terminator structure, respectively, which are all capable of target recognition. Although LhrC was originally identified as an Hfq-binding sRNA, we find that it dispenses with Hfq for the regulatory effect on *lapB*.

## MATERIALS AND METHODS

### Bacterial strains and growth conditions

The wild type strain used in this study was *L. monocytogenes* serotype 1/2c strain LO28 (27). Isogenic mutant derivatives of this strain were constructed as described previously (24). Primers used for in-frame deletions are listed in Supplementary Table S1. All strains used in the study are listed in Supplementary Table S2. In order to grow *L. monocytogenes* strains, overnight cultures were diluted 100-fold into Brain Heart Infusion medium (BHI, Oxoid) and vigorously shaken at 37°C; when appropriate, kanamycin (50 μg/ml) was added. For northern blot experiments, the following concentrations were used to induce cell envelope stress: 4 μg/ml cefuroxime (Sigma-Aldrich), 0.03% bile salts (DIFCO, No. 3), 4% NaCl, pH5 adjusted with HCl and 2% ethanol. In stress tolerance assays overnight cultures were diluted 1000-fold into BHI adjusted with cefuroxime (4 μg/ml), bile salts (0.07%), NaCl (8%), pH5 and ethanol (5%); growth was monitored for 24 h.

For cloning purposes and green fluorescent protein (GFP) reporter experiments, *Escherichia coli* TOP10 and *E. coli* DH5α (Invitrogen) were used and grown in Luria Broth (LB, Oxoid) at 37°C. When appropriate, LB was supplemented with kanamycin (50 μg/ml), ampicillin (30 μg/ml), chloramphenicol (25 μg/ml) or erythromycin (150 μg/ml).

### RNA techniques

#### Northern blot analyses

For testing the induction of LhrC in response to cell envelope stress, *L. monocytogenes* was grown to OD_600_ = 0.35, cultures were split and one of them stressed. Samples were taken from stressed and control cultures after 30 min. To assimilate an induction profile of LhrC several time points of stress were sampled (10, 20, 30, 60 and 120 min). In order to test RNA stability cells were treated with rifampicin (10 μg/ml) after 30 min of stress and samples collected at indicated time points. Cells were disrupted using the FastPrep instrument (Bio101, Thermo Scientific Corporation) and total RNA was extracted using TRI Reagent (Ambion) and purified as described previously (25). Total RNA from *E. coli* was prepared by resuspending cell pellets in 150 μl of 10 mM Na-citrate, 10 mM Na-acetate, 2 mM ethylenediaminetetraacetic acid, pH4.5. The solution was mixed with 600 μl acidic phenol and 150 μl of 10 mM Na-acetate pH4.5, 2% sodium dodecyl sulphate (SDS) and incubated at 90°C for 4 min. RNA was subsequently extracted with acidic phenol (pH4.5) and chloroform using Phase Lock Gel Heavy 2.0 ml tubes (5Prime). Supernatants were precipitated with 2.5 volumes of ethanol (96%) and 1/10 volume 3M Na-acetate (pH4.5). RNA integrity was confirmed by agarose gel electrophoresis; concentration and purity determined on a NanoDrop ND-1000 (Saveen Werner). Northern blotting of 10 μg total RNA separated on an 8% denaturing polyacrylamide gel was performed as previously described (25). DNA primers used as probes for detection of LhrC, mutated versions of LhrC, LhrA, 5S RNA of *L. monocytogenes* and *E. coli* are listed in Supplementary Table S1. RNA bands were visualized using a Typhoon Trio (GE Healthcare) and analyzed with IQTL 8.0 quantification software (GE Healthcare).

#### Reverse transcriptase-polymerase chain reaction (RT-qPCR)

Cultures were grown to OD_600_ = 0.35, split, and one stressed with 4 μg/ml cefuroxime. After 30 and 60 min samples were taken from stressed and control cultures and swiftly pelleted by centrifugation. Pellets were shock-frozen in liquid nitrogen and stored at −80°C. Total RNA was extracted according to (25). Note that 50 μg of RNA was treated with 2 μl RNAsin® (Promega) and DNase-treated according to the manufacturer's instructions (Roche). The Fermentas Maxima First Strand cDNA synthesis kit was used for cDNA synthesis on 3 μg of RNA per sample adding 0.5 μl RNAsin®. The qPCR reactions were performed using primer sets resulting in ∼100 bp long amplicons and SYBR Green PCR Master Mix (Fermentas) on a MX3000 quantitative PCR thermocycler (Stratagene) (initial step at 95°C for 10 min; 40 cycles of 15 s 95°C, 30 s 60°C and 30 s 72°C). Primers were designed using the primer design tool of Eurofins MWG Operon and are listed in Supplementary Table S1. Quantitative RT-PCR data were analyzed using the Relative Expression Software Tool—Multiple Condition Solver (REST©) version 2 (28,29). RNAs of *tpi* and *rpoB* served as reference genes. Experiments were carried out in three biological replicates having two technical replicates of each sample. Results were analyzed using two tailed Student's *t*-test. The differences reported were statistically significant with at least 95% confidence.

#### Electrophoretic mobility shift assays (EMSA)

Templates for *in vitro* transcription contained a T7 RNA Polymerase binding site at their 5′ site. In general, LhrC4 and its mutant templates were produced by PCR using overlapping primers. The template for *lapB* was prepared using primer pair T7_lapB_fw/rev on LO28 chromosomal DNA. Mutations in *lapB* were introduced by running three PCRs. PCR 1 and 2 were run on LO28 chromosomal DNA resulting in an upstream and downstream fragment of the final template, respectively. The third PCR was carried out on the products of PCR 1 and 2 using primer pair T7_lapB_fw/rev. All primers and details are listed in Supplementary Table S1. *In vitro* transcription, RNA cleaning, dephosphorylation and labeling was carried out according to (26) with minor deviations: RNA was extracted from gel by simple diffusion into 2 M NH_4_-acetate without electro elution. Dephosphorylation was achieved using shrimp alkaline phosphatase (Affymetrix Inc., USB) and labelling by polynucleotide kinase (New England Biolabs (NEB)), all in PNK buffer (NEB). Labeled transcripts were purified before gel shift employing Nucleospin miRNA kit (Macherey–Nagel) according to the manufacturer's instructions. Secondary structures of mutated LhrC4 molecules were predicted to resemble the one of wild type according to Mfold (30). The gel shifts were done according to Nielsen *et al.* (25). Briefly, 5′-end labeled LhrC4 was incubated with an indicated fold excess of non-labeled *lapB* RNA for 20 min at 37°C and additional 10 min on ice (in the presence of non-specific tRNA). For experiments testing mut_9 and mut_10, RNAs were mixed and incubated 5 min at 70°C, slowly cooled to 37°C and incubated for 40 min at 37°C before putting them on ice. Samples were loaded with current running and separated at 4°C in a 5% non-denaturing polyacrylamide gel.

#### In vitro structure probing

*In vitro* synthesized 5′ end-labeled RNAs were prepared in the same way as for EMSA experiments. Alkaline hydrolysis buffer, structure buffer, loading buffer type II, yeast tRNA and RNase T1 were from Ambion T1 RNase kit (AM2283). For an alkaline hydrolysis ladder 0.2 pmol labeled RNA was incubated in the presence of 10 μg yeast tRNA and alkaline hydrolysis buffer at 95°C for 5 min. The reaction was placed on ice and stopped by the addition of loading buffer type II. For RNase T1 sequencing ladders 0.1 pmol of labeled RNA was denatured (95°C, 1 min) in the presence of yeast tRNA (10 μg) and structure buffer. The reaction was placed at 37°C for 1 min, provided with 0.1 U of T1 RNase and incubated for another 5 min at 37°C. Structure probing of RNA-RNA interaction generally started with denaturation of 0.1 pmol labeled RNA (95°C, 1 min) which was afterwards placed on ice. In T1 RNase and lead(II)acetate experiments the labeled RNA was mixed with yeast tRNA (10 μg) and non-labeled RNA in the presence of structure buffer and kept at 37°C for 10 min. To provoke RNA cleavage the mixture was subsequently incubated with 0.1 U T1 RNase and fresh lead(II)acetate (5 mM) (Fluka), respectively. For RNase V1 or RNase A digestion, labeled denatured RNA was incubated in 10× structure buffer (37°C, 10 min) before the addition of yeast tRNA, non-labeled RNA and water. After another 10 min at 37°C, 0.05 U RNAse V1 (Ambion, AM2275) or RNase A (AM2274) was added and samples were incubated for 2 or 5 min, respectively. Details on fold excess of non-labeled RNA, amount of RNase and differing incubation times of cleaving reactions are indicated specifically. Samples were mixed with 2× loading buffer type II, denatured at 95°C for 3 min and placed on ice. A total of 2 μl of each sample was separated on an 8% polyacrylamide gel in 7 M urea at 45 W.

### Bioinformatics target prediction

The RNApredator software (31) was used to predict putative targets of LhrC1–5. All five LhrC sequences were searched against the *L. monocytogenes* EGD-e chromosome. The top five hits of each of the five result lists were chosen and merged into one final table comprising 13 putative targets (Supplementary Table S3).

### *lacZ*-fusions and β-galactosidase assays

For testing LhrC promoter activity, DNA fragments incorporating each of the five promoters were synthesized by PCR on LO28 chromosomal DNA. Resulting fragments were digested with EcoRI and BamHI and ligated as transcriptional fusions into pTCV-lac (32). The *lhrA* core promoter, as well as upstream regions of *lapB* and *lmo1669*, were also ligated into pTCV-lac, respectively. For an in-frame translational *lacZ* fusion pCK-lac was used (25). A fusion of the moderate core promoter of *lhrA* and the upstream region of *lapB* was digested with EcoRI and BamHI and ligated into the vector (resulting construct called pC-*lapB-lacZ*). All primers and details for transcriptional and translational fusions are listed in Supplementary Table S1. Beta-galactosidase assays were carried out as previously described (24). Negative β-galactosidase units were put on reference level (0 units).

### GFP reporter experiments

The RNA sequences notionally interacting were expressed in *E. coli* from two compatible plasmids, pXG-10 and pNDM220, as described in detail in (33). The *lapB* sequence was generated by PCR using primer pairs lapB_pXG10_fw/rev on chromosomal DNA of LO28, and translationally fused under constitutive expression to a *gfp* reporter gene into pXG-10 (34). LhrC4 expression was Isopropyl β-D-1-thiogalactopyranoside (IPTG) inducible from pNDM220 (35). The PCR fragment of LhrC4 was made by using primers lhrC4_pNDM220_fw/rev on chromosomal DNA. The mutated *lapB* and *lhrC4* sequences were generated in three PCRs. Details and primers are given in Supplementary Table S1. Overnight cultures of *E. coli* strains carrying both plasmids were inoculated to OD_450_ = 0.006 into fresh LB also containing ampicillin and chloramphenicol, and in case of LhrC4 induction 1 mM IPTG. Cells were grown to OD_450_ = 0.4 and harvested for western blot and northern blot analyses. One-dimensional SDS-polyacrylamide gel electrophoresis (SDS-PAGE) and subsequent western blot analysis were performed as described previously (33). The GFP (Roche) and GroEL (Sigma) monoclonal antibodies were diluted 1:10 000 and 1:50 000, respectively. Secondary mouse and rabbit horseradish peroxidase-conjugated antibodies (DakoCytomation) were diluted 1:2000. Western blots were developed using western lightning reagent (PerkinElmer) and exposed to a film (GE Healthcare). GFP signal intensity was estimated with the help of ImageJ software (36) and normalized by the corresponding GroEL signal intensities.

### LapB protein detection

LO28 wild type and Δ*lhrC1–5* were grown up to OD_600_ = 0.35, cultures split and one of each stressed with 4 μg/ml cefuroxime. After 2 h the four cultures were harvested and cell pellets washed twice in ice-cold 20 mM HEPES buffer (pH7.5). Cells were lysed in HEPES buffer containing complete protease inhibitor (Roche) using a FastPrep instrument (2 cycles of 40 s, speed 6 m/s with intermediate cooling). Extracts were centrifuged (5 min, 10 000 × *g*) and supernatants ultracentrifuged at 100 000 × *g* for 1 h. Pellets comprising the membrane protein fraction were resuspended in HEPES buffer with the help of a hand homogenizer (VWR) and protein concentration determined via a BCA assay (PIERCE) according to the manufacturer's instructions. Western blot analysis was performed as described earlier (33) with the following modifications: 60 μg of proteins were separated via 1-D SDS-PAGE which was run at 100 V for 3 h to ensure the large LapB protein (185 kDa) migrated into the gel. Proteins on the polyvinylidene difluoride (PVDF) membrane were stained with amido black staining solution (1% acetic acid, 10% methanol, 0.1% (w/v) Naphthol Blue Black (Santa Cruz Biotechnology, Inc.) which served as a loading control. After destaining, the membrane was incubated with polyclonal α-LapB antibody (1:3000) generously provided by Didier Cabanes (37). Rabbit horseradish peroxidase-conjugated secondary antibody was diluted 1:2000 (DakoCytomation). The relative amount of LapB was estimated by using ImageJ (36). LapB signals were normalized by means of a representative band of the amido black staining.

## RESULTS

### The role of LhrC1–5 during cell envelope stress

**Figure 1.**
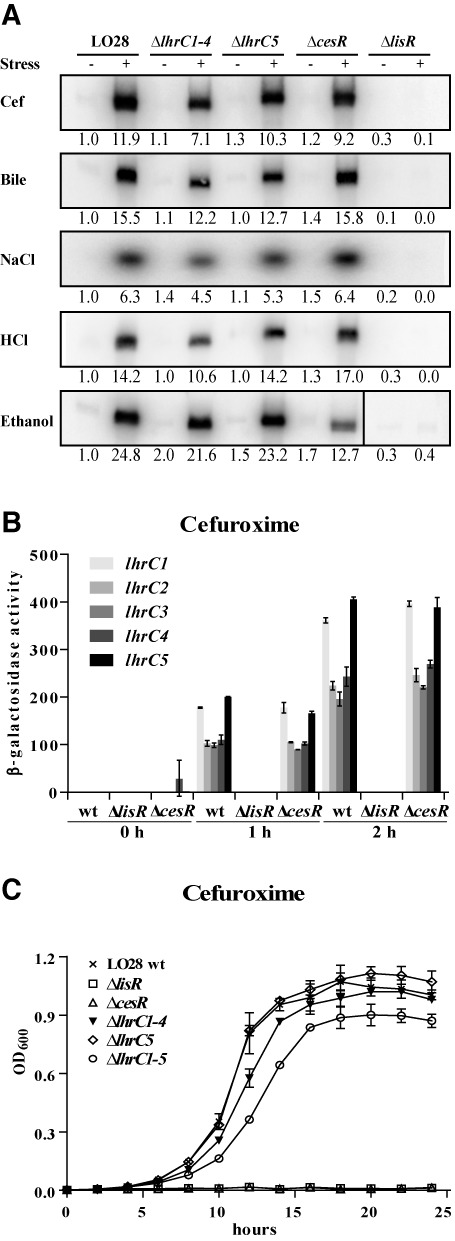
The role of LhrC1–5 during cell envelope stress. (**A**) Northern blot analyses of LhrC expression. Samples were taken from *L. monocytogenes* LO28 wild type (lane 1, 2), Δ*lhrC1–4* (3,4), Δ*lhrC5* (5,6), Δ*cesR* (7,8) and Δ*lisR* (9,10) from cultures stressed with various agents acting on the cell envelope (+) as well as from control samples without stress (−). Northern blots were probed for LhrC1–5 and 5S rRNA as a loading control (Supplementary Figure S2A). Relative levels of LhrC (normalized to 5S) are shown below each lane. (**B**) Transcriptional reporter gene fusions of *lhrC* promoters. Promoter regions of all five *lhrC* copies were each cloned into vector pTCV-lac and plasmids transformed into LO28 wild type, Δ*lisR* and Δ*cesR*. The resulting 15 strains were grown up to OD_600_ = 0.35 and stressed with cefuroxime (4 μg/ml), after control samples had been taken (0 h). Further samples for a following β-gal assay were withdrawn after 1 and 2 h of ongoing stress. Results are the average of two biologically independent experiments, each carried out in technical duplicates. (**C**) Stress tolerance assay. Wild type, Δ*lhrC1–4*, Δ*lhrC5*, Δ*lhrC1–5*, Δ*lisR* and Δ*cesR* were grown in BHI containing 4 μg/ml cefuroxime. Bacterial growth was monitored for 24 h. The average of three independent biological replicates is shown.

Induction of the five LhrC sRNAs (LhrC1–5) was recently reported for *L. monocytogenes* residing in human blood (6) as well as inside macrophages (11). In order to determine the exact physical conditions leading to LhrC1–5 expression, *L. monocytogenes* was exposed to subinhibitory concentrations of numerous stresses, and LhrC1–5 levels determined via northern blot analysis. The sRNAs were found to be induced in LO28 wild type by a whole range of stress agents affecting the integrity of the cell envelope, including the β-lactam antibiotic cefuroxime, bile salts, high osmolarity, low pH and ethanol (Figure 1A, Supplementary Figure S2A). Due to their virtual sequence identity, the probe used for northern blotting binds to all five LhrC copies. An LhrC signal was still detected in a mutant lacking *lhrC5* as well as in one lacking the *lhrC1–4* locus meaning that both loci were expressed during cell envelope stress (Figure 1A). The signal was lost after knockout of all five *lhrC* copies (Supplementary Figure S2B) proving the specificity of the northern blot probe and a full *lhrC* mutant to be actually on hand.

The promoter activity of the single *lhrC* copies was determined using transcriptional fusions of each of the five *lhrC* promoters to the reporter gene *lacZ* in vector pTCV-lac (32). Prior to the addition of cefuroxime, no β-galactosidase (β-gal) activity was detected in wild type cells harboring any of the five *lhrC-lacZ* fusion plasmids, i.e. all five *lhrC* promoters were inactive or transcription was beneath the detection limit of the assay (Figure 1B). The activity of all five promoters increased dramatically when cell surface integrity was disturbed by cefuroxime with promoters *lhrC1* and *lhrC5* being most strongly induced, relative to the pre-stressed condition (Figure 1B). While the extent of induction was different, the induction pattern among the five promoters was the same when exposing the cells to ethanol or bile salts stress (Supplementary Figure S3).

In order to determine the importance of LhrC1–5 for stress tolerance, growth of the wild type and mutant strains Δ*lhrC1–5*, Δ*lhrC1–4* and Δ*lhrC5* was assessed under stress conditions known to affect the integrity of the cell envelope (cefuroxime, ethanol, bile salts, low pH, high osmolarity). Previous studies have shown that *L. monocytogenes* defective in the TCSs LisRK or CesRK shows growth defects in the presence of several such stressors (38–42). As a control, mutants lacking the response regulators LisR or CesR were carried along in the stress tolerance assays. No difference in growth could be observed between wild type and the three *lhrC* mutant strains when exposed to acid, ethanol or osmotic stress conditions (Supplementary Figure S4). However, in the presence of 4 μg/ml cefuroxime the Δ*lhrC1–5* mutant strain exhibited a defect in growth compared to wild type (Figure 1C). Interestingly, also Δ*lhrC1–4* was hampered, yet not to the same extent as the full mutant. As expected, mutants lacking the response regulators LisR or CesR were not able to grow in 4 μg/ml cefuroxime (Figure 1C). A similar tendency was observed when cells were exposed to 0.07% bile salts (Supplementary Figure S4).

### The TCS LisRK is mandatory for LhrC expression

Because LhrC proved to be strongly induced by a series of cell envelope acting agents, and a knockout of *lhrC1–5* resulted in phenotypes that to some extent resembled the ones observed for mutants in the TCSs LisRK and CesRK, we considered the possibility that the sRNAs LhrC1–5 are controlled by at least one of these TCSs. Therefore, mutants lacking the response regulators LisR and CesR, respectively, were investigated for the presence of LhrC1-5 during cell envelope stress in northern blot analyses (Figure 1A). In a Δ*lisR* mutant background, no LhrC1–5 could be detected at any of the stress conditions tested, meaning that a functional LisRK TCS is mandatory for LhrC expression. The picture of Δ*cesR* resembled the one of wild type with the exception of ethanol stress. Here, the LhrC signal was weaker in Δ*cesR* compared to wild type indicating an involvement of the CesRK TCS in LhrC1–5 expression for this specific stress condition. However, LisRK was still essential for LhrC1–5 expression during ethanol stress (Figure 1A). Further, activity of the five *lhrC* promoters was tested in a Δ*lisR* and Δ*cesR* background using reporter gene fusions. In a Δ*lisR* background, all five *lhrC* promoters were inactive for the tested conditions (cefuroxime, bile salts and ethanol) (Figure 1B, Supplementary Figure S3). The Δ*cesR* strain resembled wild type in the presence of cefuroxime and bile salts, but stressed with ethanol, Δ*cesR* cells expressed a reduced level of all five LhrC copies, confirming the observation made on northern blots. In conclusion, the TCS LisRK is mandatory for transcription of all five LhrC copies, whereas CesRK is also involved in LhrC1–5 induction during ethanol stress.

**Figure 2.**
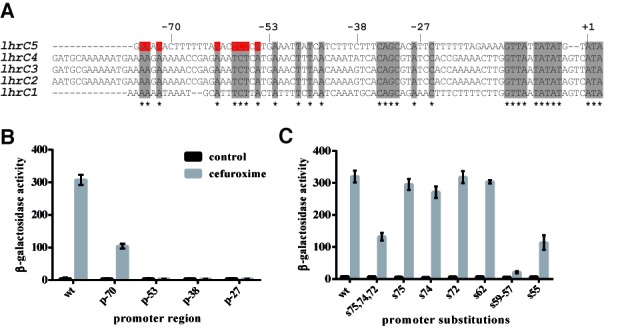
The promoter region of *lhrC5* contains a LisR-responsive element. (**A**) Alignment of the five *lhrC* promoters. The promoter sequences were aligned using ClustalW 2.0 (47). Conserved nucleotides are marked in gray. Conserved nucleotides substituted in mutant derivatives of the *lhrC5* promoter are shown in red. Numbers refer to *lhrC5*; +1 corresponds to the transcriptional start site. (**B**) Transcriptional reporter gene fusions of truncated *lhrC5* promoter. Promoter regions from −70, −55, −38 and −28 to + 87 relative to the *lhrC5* transcriptional start site were fused to a promoter-less *lacZ* gene in pTCV-lac. The complete intergenic region between the upstream gene (*lmo0946*) and *lhrC5* was used as a positive control (wt). Samples for β-gal assays were taken from cultures stressed with cefuroxime for 1 h and from corresponding control samples. The presented β-gal activities are the average of three independent experiments each conducted in duplicates. (**C**) Transcriptional reporter gene fusions of *lhrC5* promoter mutants. The *lhrC5* promoter was substituted at indicated positions and such mutated promoters tested as described in (B).

Many sRNAs have been reported to be regulated by TCSs in Gram-negative bacteria (43), and also for some Gram-positive bacteria with a low GC-content, an interconnection of TCSs and sRNAs has been described (44–46). LhrC represents the first sRNA in *L. monocytogenes* described to be regulated by a TCS. Despite intensive research on the LisRK system (38,40,42), no consensus sequence for LisR binding in promoter regions of LisRK-regulated genes has been identified yet. In order to pinpoint the regions in the five *lhrC* promoters that are important for LisR-dependent induction, promoter sequences of the five *lhrC* copies were aligned using ClustalW 2.0 (47), and conserved nucleotides, which might constitute a LisR-responsive element, were determined (Figure 2A). In a next step, regions of the *lhrC5* promoter were successively deleted to narrow down the LisR-responsive element. The cropped versions of the *lhrC5* promoter were analyzed in transcriptional fusion experiments as depicted in Figure 2B. Results revealed the promoter region upstream of -53 from the transcriptional start site to be important for LhrC induction. In the following, conserved nucleotides upstream of −53 were substituted and the ability of the mutated promoters to be induced tested via transcriptional fusions (Figure 2C). Substitution of nucleotides −57 to −59 almost entirely abolished induction of *lhrC5-lacZ*, but also a single substitution of position −55 and a concurrent change in sites −72, −74 and −75 had a negative effect on induction. These residues most likely reside within the LisR-responsive element important for induction of LhrC1–5 in response to cell envelope stress.

### The *lapB* mRNA is target of LhrC

**Figure 3.**
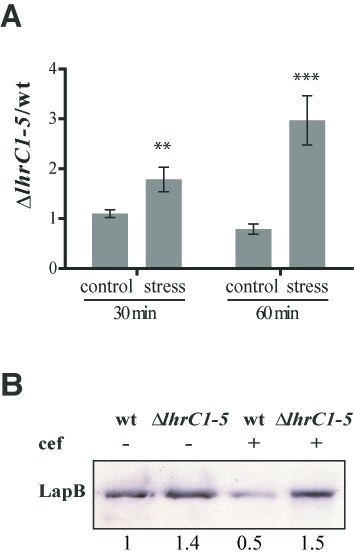
Expression of *lapB* is controlled by LhrC1–5. (**A**) LhrC-mediated down-regulation of *lapB* mRNA. *lapB* mRNA was quantified in Δ*lhrC1–5* relative to wild type (wt) by RT-qPCR. The ratio of Δ*lhrC1–5*/wt was determined at the time points 30 and 60 min for both non-stressed (control) and cefuroxime-stressed samples. The result shown is the average of three independent experiments each conducted in duplicate. Two and three asterisks indicate a significant increase of the ratio under stress conditions compared to the corresponding control with *P* < 0.005 and *P* < 0.001, respectively. (**B**) LhrC-mediated down-regulation of LapB protein. For western blot analysis of LapB protein, samples were taken 2 h post-treatment with cefuroxime (4 μg/ml) from wild type (wt) and Δ*lhrC1–5*, but also from unstressed cultures. Surface protein enriched extracts were separated via 1D-PAGE, and LapB protein was detected using an α-LapB antibody. As a loading control, all proteins on the membrane were stained with amido black (Supplementary Figure S6B). Relative levels of LapB (normalized to control) are shown below each lane.

A bioinformatics approach was pursued to search for putative direct RNA-targets of LhrC1–5 using the software RNApredator (31). All five copies of LhrC were included in this search. A list of targets containing the five top hits for each LhrC copy was built resulting in a number of 13 putative targets in total (Supplementary Table S3). Assuming that action of LhrC1–5 on an mRNA target will change the abundance of the latter one, real-time RT-qPCR was employed to survey for changed mRNA levels of the 13 putative targets in Δ*lhrC1–5* after cefuroxime stress. For this purpose cultures of wild type and Δ*lhrC1–5* cells were grown up to OD_600_ = 0.35, split, and one of them stressed with cefuroxime. Samples for RNA purification were taken from unstressed and stressed cultures 30 min and 1 h after onset of stress. These time points were chosen in conclusion from a time course experiment disclosing the strongest LhrC1–5 induction 1 h after cefuroxime had been added (Supplementary Figure S5). Detailed RT-qPCR results of two biological replicates on all 13 targets are provided in Supplementary Table S4. A third biological replicate was performed on four of the targets: *lapB* (formerly known as *lmo1666*), *lmo1041*, *lmo1993* and *actA* (Figure 3A, Supplementary Figure S6A). Figure 3A shows the ratio of mutant and wild type for the target with the most significant difference between control conditions (LhrC1–5 absence) and cefuroxime stress (LhrC1–5 presence): The *lapB* mRNA encoding a cell wall protein recently identified as a virulence determinant in *L. monocytogenes* (37). *lapB* mRNA was approximately three times as abundant in the mutant after 1 h of cefuroxime stress, but not under control conditions. Notably, of the initially predicted 13 targets, *lapB* was the only one ranked in top 5 of all five LhrC copies (Supplementary Table S3).

As an implication of the results obtained from RT-qPCR experiments, one would assume reduced amounts of LapB protein in times of high LhrC1–5 expression. In order to evaluate the effect of LhrC1–5 on LapB protein level, a western blotting experiment was performed. Surface proteins of *L. monocytogenes* were prepared from wild type and Δ*lhrC1–5*, both from cultures stressed with cefuroxime and from unstressed samples. Cefuroxime stress was exerted on the cells for 2 h justified by the peak expression of LhrC1–5 not before 1 h of stress (Supplementary Figure S5). Whereas LapB levels in unstressed samples were comparable between the wild type and Δ*lhrC1–5*, there was a clear down-regulation of the protein in the wild type strain in response to cefuroxime stress but not in the LhrC mutant (Figure 3B, Supplementary Figure S6B). Thus, induction of LhrC during cell envelope stress indeed has a verifiable impact on LapB quantity.

### Direct interaction of LhrC4 and *lapB* mRNA

**Figure 4.**
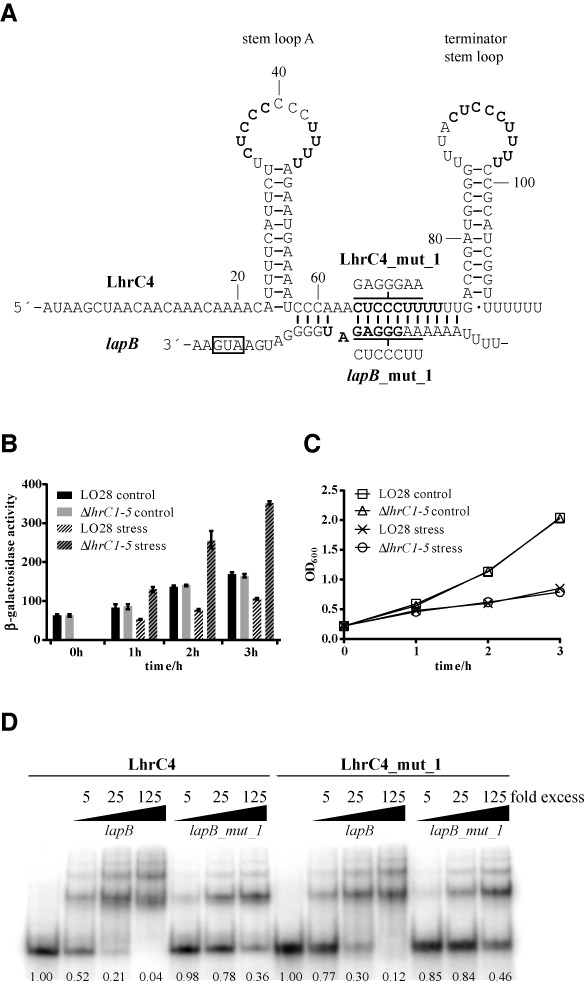
Predicted LhrC and *lapB* mRNA interaction. (**A**) According to the RNApredator software the single-stranded stretch between the two stem loops of LhrC binds to *lapB* mRNA thereby blocking the Shine–Dalgarno sequence (shown in bold). LhrC4 is shown as a representative of the five LhrC copies. Three similar sequences CUCCC(…)UUUU in loop A, the single-stranded stretch and the terminator loop, repectively, are marked in bold. The sequences mutated in LhrC4_mut_1 and *lapB_mut_1* are shown. Lines above and below LhrC4 and *lapB*, respectively, indicate the regions mutated in the mut_1 variants. (**B**) LhrC-mediated down-regulation of *lapB-lacZ* expression. β-gal activities were assessed in wild type and Δ*lhrC1–5* strains carrying pC-*lapB-lacZ*. Under non-stress conditions β-gal activity of wild type and mutant was comparable at all tested time points. After cefuroxime stress, β-gal activity in the wild type strain decreased whereas in Δ*lhrC1–5* an increase was observed. After 2 and 3 h of stress, β-gal activity in the mutant was more than three times as high as in the wild type, pointing to a released negative regulation in Δ*lhrC1–5*. Results of the β-gal assay are the average of four biological replicates each conducted in technical duplicates. (**C**) Growth of Δ*lhrC1–5* and wild type strains, tested in the β-gal assay shown in Figure 4(B), was comparable at all tested time points. (**D**) Gel mobility shift assay of the interaction between *lapB* mRNA and LhrC4. Labeled LhrC4 was shifted with increasing concentrations of *lapB* mRNA. Mutation of 7 nt in the *lapB* sequence predicted to be involved in the interaction (*lapB_mut_1*) reduced the interaction. Correspondingly mutated LhrC4 (LhrC4_mut_1) was still capable of *lapB* mRNA binding and could not compensate for the mutation in *lapB_mut_1*. The fraction of unbound LhrC is shown below each lane.

A reporter gene fusion strategy was employed to provide evidence for a post-transcriptional effect of LhrC1–5 on *lapB*. The RNApredator software predicted an interaction of a single stranded stretch of LhrC1–5 and the ribosome binding site of *lapB* mRNA (Figure 4A). The gene encoding LapB is the last in an operon of four genes (*lmo1669*, *lmo1668*, *lmo1667* and *lmo1666* (*lapB*)) according to the *L. monocytogenes* operon structure (6). In addition, Reis *et al.* reported PrfA-dependent *lapB* transcription from a promoter directly upstream of *lapB* (37). However, neither a fusion of the 400 bp region upstream of *lapB* to a promoter-less *lacZ*, nor a fusion of the upstream region of *lmo1669* (300 bp) to *lacZ*, yielded any activity in β-gal assays in our hands (Supplementary Table S5). Notably, *lapB* mRNA could be detected in RT-qPCR experiments, so the β-gal assay was assumed to be not sensitive enough to report transcription of *lapB* from its natural promoters at the tested conditions. To still make use of a reporter gene fusion strategy, the intergenic *lmo1667-lapB* sequence (100 bp) and additional 32 bp of *lapB*'s coding region were fused downstream to a moderate promoter, and in-frame to *lacZ* in the translational reporter vector pCK-lac (26) (Supplementary Figure S7). Comparison of this construct (pC*-lapB-lacZ*) in wild type and Δ*lhrC1–5* cells showed increased β-gal activity in the mutant after cefuroxime stress (more than 3-fold), but not under non-stress conditions (Figure 4B and C). This clearly indicates that the region −100 to +32 of *lapB* includes a sequence mediating decreased expression of *lapB* after cefuroxime stress, but only when LhrC1–5 is present. A difference in transcription of the moderate promoter in wild type and Δ*lhrC1–5* was excluded by carrying along its transcriptional fusion to *lacZ* (Supplementary Figure S8). The Δ*lhrC1–4* and Δ*lhrC5* cells were also transformed with pC-*lapB-lacZ*, and β-gal activity measured under control conditions and after cefuroxime stress (Supplementary Figure S9). None of the mutants showed an increase in translation as observed for Δ*lhrC1–5*, but in both mutants β-gal activity increased compared to the wild type after cefuroxime stress, only slightly in Δ*lhrC5*, but clearly in Δ*lhrC1–4*.

To further substantiate the predicted direct interaction of *lapB* mRNA and LhrC, without the involvement of any other factors in *Listeria*, both RNAs were expressed from two compatible plasmids in *E. coli* (33). The *lapB* sequence (from −100 to +32 relative to translational start) was expressed from a constitutive promoter in pXG-10, a vector featuring GFP as reporter (34). Expression of the sRNA was induced by IPTG from plasmid pNDM220 (35). Of the five existing LhrC copies, LhrC4 was predicted to bind *lapB* mRNA with the highest energy of interaction (−18.48 kJ/mol) according to RNApredator, and was therefore chosen for cloning into pNDM220. Induction of LhrC4 led to a reduction of the GFP signal on a western blot (Supplementary Figure S10A, lane 4) indicating an interaction of the two RNAs in this *E. coli* reporter system. In order to ensure that reduction of the GFP signal indeed resulted from binding of LhrC4 to *lapB* mRNA, the *lapB* sequence was mutated to disrupt interaction with LhrC4 (Supplementary Figure S10B). The mutation reduced the negative effect LhrC4 had on GFP expression (Supplementary Figure S10B, lane 5). Curiously, the interaction of the two RNAs could not be disrupted by expressing wild type *lapB* and correspondingly mutated LhrC4 (Supplementary Figure S10B, lane 6). A similar expression level of LhrC4 and mutated LhrC4 in *E. coli* was assured by detecting both versions of the sRNA via northern blot analysis (Supplementary Figure S10B). A gel mobility shift experiment was consulted as another independent method to clarify the interesting discrepancy that relieving the proposed RNA-RNA interaction worked only unidirectionally. The gel mobility shift (Figure 4D) validated this observation leading to the conclusion that *lapB* was indeed mutated in the sequence interacting with LhrC4, but that another region than the predicted one of LhrC4 must be involved in *lapB* mRNA binding.

*In vitro* binding experiments were also employed to test whether Hfq presence could stimulate the association of the two RNAs (Supplementary Figure S11). These gel shift experiments demonstrated the ability of LhrC4 to bind Hfq, but with an increasing concentration of *lapB* mRNA, the presence of Hfq had no effect on sRNA-mRNA complex formation. Moreover, the translational fusion vector pC*-lapB-lacZ* in a Δ*hfq* background gave rise to a β-gal activity equivalent to wild type (Supplementary Figure S12). Finally, LhrC did not require Hfq in terms of stability in strain LO28 (Supplementary Figure S13). Collectively, these results demonstrated that Hfq is dispensable for the regulatory action of LhrC on *lapB*.

### The multiple faces of LhrC

**Figure 5.**
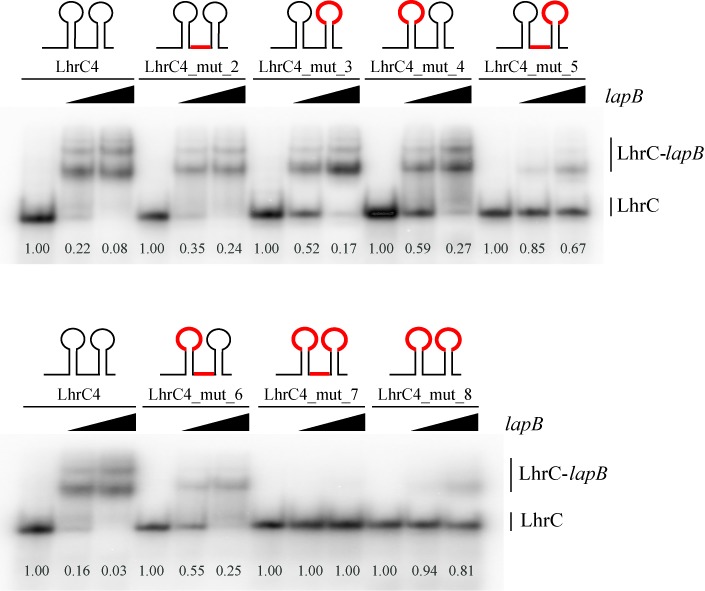
LhrC4 mutant screening of loop A, single-stranded stretch, and the terminator loop. Labeled LhrC4 and several mutant derivatives were tested for their ability to interact with *lapB* RNA (25 and 125 times excess). In the LhrC sketches, mutated regions are shown in red. LhrC4: wild type LhrC4. LhrC4_mut_2: mutation in single-stranded stretch. LhrC4_mut_3: mutation in terminator loop. LhrC4_mut_4: mutation in loop A. LhrC4_mut_5: mutation in single-stranded stretch and terminator loop. LhrC4_mut_6: mutation in single-stranded stretch and loop A. LhrC4_mut_7: mutation in single-stranded stretch, loop A and terminator loop. LhrC4_mut_8: mutation in loop A and terminator loop. The sequences of the LhrC mutants are shown in Supplementary Figure S14. The fraction of unbound LhrC is shown below each lane.

A closer inspection of the sequence of LhrC4 revealed similarity of the sequences of the two loops and the proposed site of interaction in the single-stranded stretch between the two stem loops (Figure 4A). The sequence 5’-CUCCC and a series of four Us can be found at all three sites in the sRNA, holding perfect complementarity with *lapB*. As loops have been extensively described as being best-suited for an initial interaction of two RNAs (48–50), we took both loops (loop A and terminator loop) into consideration to be the actual site of interaction with *lapB*. Several different LhrC4 mutants were screened for their ability to bind *lapB* mRNA in gel mobility shift experiments (Figure 5). The sequences of these LhrC mutants are shown in Supplementary Figure S14. Mutation of the single-stranded stretch (LhrC4_mut_2) did not reduce any of the interaction ability of the two RNAs. In contrast, mutation of the terminator loop (LhrC4_mut_3) or loop A (LhrC4_mut_4) indeed had a negative effect on LhrC4-*lapB* mRNA binding. Mutating both the terminator loop and the single-stranded stretch (LhrC4_mut_5) disturbed sRNA-mRNA interaction even more than the sole mutations of the terminator loop (LhrC4_mut_3) and the single-stranded stretch (LhrC4_mut_2). Mutating both loop A and the single-stranded stretch (LhrC4_mut_6) resulted in a binding resembling that of the sole loop A mutant to *lapB* mRNA (LhrC4_mut_4). When both loops of LhrC4 were mutated in combination (LhrC4_mut_8) there was almost no detectable binding to *lapB* mRNA. Eventually, the concerted mutation of all three homologous sites in LhrC4 (LhrC4_mut_7) entirely abolished interaction with the target RNA. In conclusion, both loops of LhrC4 as well as the single-stranded stretch are capable of mediating binding to *lapB* mRNA, however, the capability of the latter seems to be much smaller compared to the loops.

**Figure 6.**
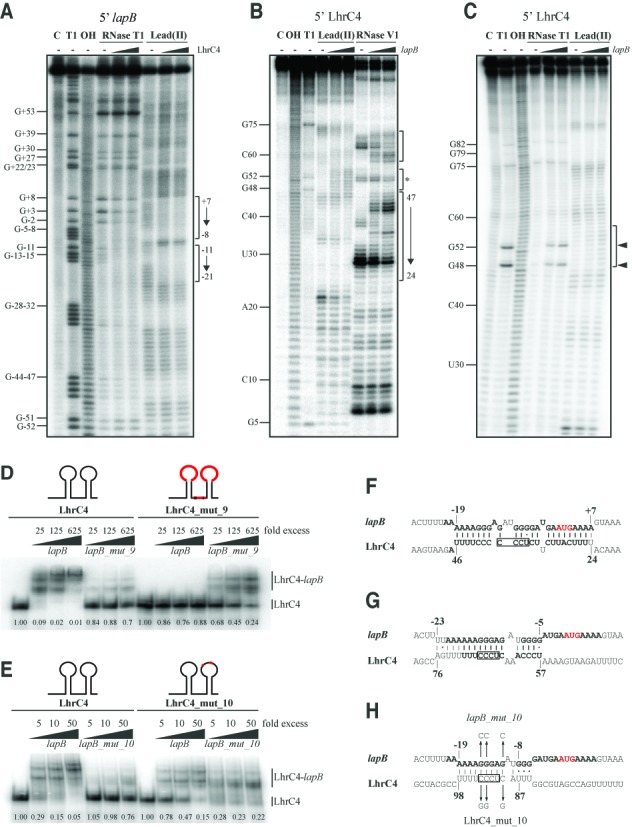
(**A**), (**B**) and (**C**): Structure probing of LhrC4 and *lapB* mRNA interaction. (**A**) 5′ labeled *lapB* mRNA was partially digested with RNase T1 (5 min) or cleaved by lead(II) (2 min), either in the absence (−) or in the presence of 25- or 125-fold excess of LhrC4. As a control, untreated *lapB* mRNA was separated (C, lane 1). An RNase T1 ladder (T1) was separated in lane 2, and cleaved G residues are labeled along the left side of the gel. An alkaline ladder (OH) is shown in lane 3. The *lapB* sequence found to be protected from cleavage during LhrC4 presence is indicated on the right side of the gel. (**B**) Partial cleavage of 5′ labeled LhrC4 with lead(II) for 1 min (lanes 4–6) or RNase V1 for 2 min (lanes 7–9). LhrC4 was either digested alone (−) (lanes 4 and 7) or in the presence of 125- or 500-fold excess of non-labeled *lapB* mRNA. Untreated LhrC4 (C), an alkaline ladder (OH) and an RNase T1 ladder (T1) are shown in lane 1, 2 and 3, respectively. For an overview, selected nucleotides are labeled on the left side. Sequences showing structural changes upon *lapB* binding are marked on the right side of the gel: Stem loop A (lower and middle brackets; the 3′ side of stem A is marked by an asterisk) and the single-stranded stretch (upper bracket). (**C**) Labeled LhrC4 was partially cleaved with RNase T1 for 3 min (lanes 4–6) and lead(II) for 1 min (lanes 7–9) in the absence (−)(lanes 4 and 7) or presence of increasing amounts of non-labeled *lapB* mRNA (25-fold excess in lanes 5 and 8; 125-fold excess in lanes 6 and 9). Untreated LhrC4 (C), an RNase T1 (T1) and alkaline ladder (OH) are shown in lane 1, 2 and 3, respectively. On the left, the location of selected nucleotides is labeled. On the right, the observed change in secondary structure in the 3′ side of stem A upon *lapB* mRNA binding is marked (arrows: G48 and G52). (**D**) and (**E**) Gel mobility shift assays. In the LhrC sketches, mutated regions are shown in red. (**D**) *lapB_mut_9* was mutated in the sequence interacting with LhrC4 and lost almost all of its capability to shift LhrC4. LhrC4_mut_9, the sRNA compensatory mutated in loop A, single-stranded stretch and terminator loop, was not shifted by *lapB* mRNA, but could partially compensate for the mutation in *lapB_mut_9*. An overview of the substitutions in *lapB_mut9* and LhrC_mut_9 is shown in Supplementary Figure S17. Numbers in the bottom indicate the fraction of LhrC4 that was not shifted. (**E**) Mutation of only three sites in the *lapB* sequence (*lapB_mut_10*) nearly abolished all of the binding between the two RNAs. LhrC4_mut_10 which carried a corresponding mutation only in the terminator loop could still be shifted by *lapB*, but was also able to partially compensate for the mutation in *lapB_mut_10*. (**F**), (**G**) and (**H**) Deduced base pairing of *lapB* mRNA and loop A, the single-stranded stretch and the terminator loop of LhrC4, respectively. The *lapB* mRNA and LhrC4 sequences found to be bound in structure probing experiments is printed in bold. The start codon is marked in red. The UCCC motif is boxed. In figure (**H**) the three point mutations of mut_10 are indicated.

In order to further explore the binding of LhrC4 to *lapB* mRNA, structural probing was employed on 5′ labeled *lapB* mRNA as well as on 5′ labeled LhrC4. RNA cleavage of *lapB* mRNA was provoked by RNase T1 at single-stranded G residues or lead(II) acetate treatment at single-stranded sequences in general. The *lapB* mRNA sequence originally predicted to be involved in LhrC4 binding (−21 to −7 relative to translational start) could be confirmed by RNA probing, since it was partially protected from cleavage in the presence of LhrC4 (Figure 6A; from −21 to −11). But surprisingly, the interaction was not limited to this site. Protection from cleavage stretched out down to position +7, into the coding region of *lapB* (Figure 6A; from −8 to +7).

**Figure 7.**
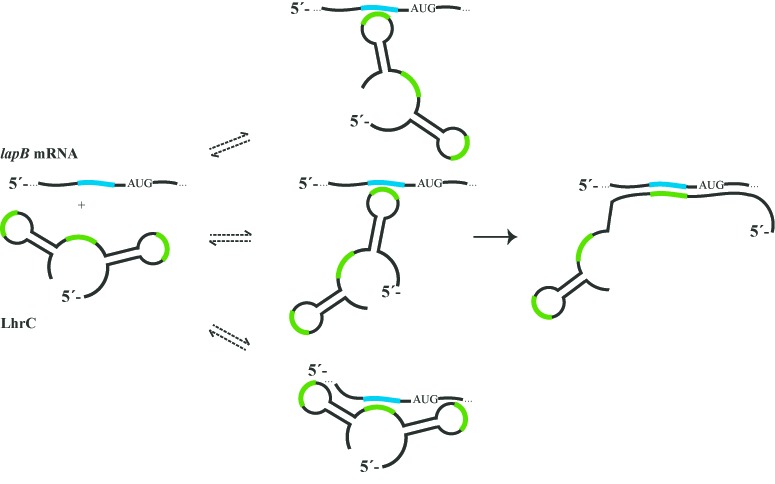
Model of LhrC4-*lapB* mRNA interaction. Three different sites in LhrC4, all containing a UCCC motif (green), are capable of binding to the G-rich sequence (blue) upstream of the start codon in *lapB*. Formation of the final sRNA-mRNA duplex involves opening of stem loop A, i.e. the 5′ side of stem A and loop A are bound to *lapB* mRNA. One LhrC4 molecule could potentially bind two or three *lapB* mRNA molecules simultaneously (not illustrated).

Because the sRNA LhrC is poor in guanosine residues, cleavage of 5′ labeled LhrC4 was not only induced by RNase T1 (cuts single-stranded G) and lead(II) treatment, but additionally by RNase V1 cleaving double-stranded RNA and single-stranded stacked RNA. Structural probing of unbound LhrC4 revealed that loop A might exist in the form proposed in Figure 4A, but an alternative structure is also conceivable, as cleavage by RNase V1, but not lead(II), was observed in the UCCC sequence in loop A (Figure 6B and C). Interestingly, a change in secondary structure of stem A was detected when the sRNA was incubated with *lapB* mRNA (Figure 6B and C): In the 3′ side of stem A, position 48–56 changed from bound- to single-stranded status, whereas position 24–32 in the 5′ side of stem A remained in a double-stranded structure. Remarkably, position 24–32 in stem A exhibits perfect complementarity to the *lapB* sequence from −2 to +7 leading to the conclusion that these two sequences interact, as illustrated in Figure 6F. Regarding the three sites of LhrC4 that were determined by gel shift experiments to be important for *lapB* mRNA interaction: Lead(II) treatment did not show a change in secondary structure in any of the two loops or in the single-stranded stretch of LhrC4, however, RNase V1 could clearly reveal loop A to change from unbound- to double-stranded state after addition of *lapB* mRNA, and also the stretch between the loops was subject to a change in secondary structure (Figure 6B). The matching interactions between these two sites in LhrC4 and *lapB* mRNA are illustrated in Figure 6F and G, respectively. The sequence of the terminator loop was not covered in this 5′ labeling experiment, however, its role in binding to *lapB* mRNA will be specifically addressed in gel shift experiments presented below. From the results obtained until this point we assumed that any of the three homologous sites in LhrC4 can make the first contact with the complementary sequence in *lapB* mRNA (−19 to −11) before a structural rearrangement in the sRNA takes place and the final sRNA-mRNA duplex forms involving stem loop A in LhrC4 and the sequence −21 to +7 of *lapB* (Figure 7, Supplementary Figure S15). For an intended mutation of LhrC4 to destroy interaction of the two RNAs with a subsequent compensatory mutation in *lapB*, the stem A sequence of LhrC4 seemed to be best suited and more straightforward than picking a sequence in *lapB* mRNA that is capable of an interaction with three different sites in LhrC4. Hence, stem A was mutated by inversion of the sequence (5′ ≥ 3′ to 3′ ≥ 5′) to keep the structure of LhrC4 (Supplementary Figure S16). However, mutation of stem A did not result in any reduction of binding efficiency of the two RNAs *in vitro* (Supplementary Figure S16). This observation endorsed our theory that the stem opening and binding represents a later event in the course of sRNA-mRNA complex formation, meaning that the two RNAs are still capable of binding, but the final duplex cannot be formed. In an attempt to still mutate and compensate, *lapB* was mutated (*lapB_mut_9*) to prevent most of its interaction with LhrC4. Likewise, all three putative interaction sites in LhrC4 were substituted to match *lapB_mut_9* (LhrC4_mut_9) (Supplementary Figure S17). The interaction capability of *lapB_mut_9* and LhrC4_mut_9 could not be fully restored to wild type levels, but the *lapB* mutation could indeed be compensated for partially by the mutations in all three LhrC4 interaction sites (Figure 6D).

The three sites in LhrC4 important for *lapB* mRNA binding all contain a UCCC motif which was reported in the literature to be a common loop motif for target recognition in sRNAs of several Gram-positive bacteria (51). We wondered whether this motif mediates first contact between LhrC4 and *lapB* mRNA as well. In order to test this, the target *lapB* was mutated in two nucleotides in the site where the UCCC motif of LhrC4 is predicted to bind, in addition to a single nucleotide flanking this site (5′-GGGAG to 5′-CCGAC), which was mutated to keep the secondary structure of the compensatory LhrC4 mutant mentioned below. Strikingly, this minimal mutant (*lapB_mut_10*) lost almost all of its binding capability to LhrC4 demonstrating the importance of the implied motif for target recognition (Figure 6E and H). A compensatory LhrC4 mutant (LhrC4_mut_10) was constructed, but this time mutating only the according three nucleotides in the terminator loop (5′-CUCCC to 5′-GUCGG) to unequivocally demonstrate its involvement in *lapB* mRNA binding (Figure 6H). LhrC4_mut_10 lost only part of its ability to bind *lapB* mRNA, once more indicating that there are two more possible sites in the sRNA capable of an interaction with one important binding site in the mRNA (Figure 6E). The compensatory terminator loop mutations indeed regained the ability of LhrC4 to shift *lapB_mut_10*, although not to the same level of binding as seen for *lapB* wild type and LhrC4 wild type (Figure 6E), since LhrC4_mut_10 features only a single site that can bind to *lapB_mut_10* instead of three sites (corresponding to the wild type situation).

## DISCUSSION

### Induction of LhrC

This study aimed to characterize the five homologs of LhrC which were repeatedly found to be highly induced under infection-relevant conditions (6,11). We show that disturbance of the cell envelope integrity is the actual signal for LhrC1–5 induction, and that the presence of the sRNAs contributes to the growth of *L. monocytogenes* during cell envelope stress. An involvement of sRNAs when dealing with cell envelope stress has been frequently described for Gram-negative bacteria. In fact, a disproportionately high number of trans-encoded sRNAs regulate proteins of the outer membrane (OMP) or transporters (1,52) which can to some extent be explained by the relatively easy detection of these highly abundant proteins. Although the cell envelope stress response of Gram-positive bacteria is a long and intensively studied research area (53), reports on an involvement of sRNAs are rare in this respect. Many of the numerous OMP regulating sRNAs in *E. coli* are aptly regulated by cell envelope stress sensing systems (54,55). Analogously, we checked the possibility of LhrC1–5 being regulated by one of the stress sensing systems of *L. monocytogenes* and could demonstrate that the TCS LisRK is mandatory for LhrC induction. This poses LhrC as the first sRNA in *L. monocytogenes* to be regulated by a TCS. A role of LisRK in the stress response to low pH and high ethanol concentrations has been described long ago (38), just as its importance for *L. monocytogenes’* virulence (38,39). Later, LisRK was reported to also be involved in osmoregulation (56) and in dealing with the lantibiotic nisin as well as cephalosporin antibiotics (40). Finally, a comprehensive study by Nielsen *et al.* unveiled LisR as a central regulator controlling a whole series of genes after cell envelope stress (42). With LhrC being absolutely dependent on the LisRK system, one could speculate about the sRNA to be a hitherto unknown regulatory component between LisR and its responsive genes. Small RNAs being regulated by a TCS are capable of controlling a major fraction of the TCS’ regulon which is maxed out by the GacAS system of *P. aeruginosa* acting exclusively through the sRNAs RsmY and RsmZ (57).

### *LapB* mRNA is target of LhrC

In the scope of our work we could identify *lapB* mRNA as the first direct target of the sRNAs LhrC1–5. The gene *lapB* was predicted to have a σ^B^-dependent promoter 67 bp upstream of its translational start site (58) and its expression was reported to be positively influenced by the major listerial transcriptional activator of virulence genes PrfA (37,59). Both σ^B^ regulation as well as positive control by PrfA point to an involvement of LapB in stress response and/or virulence. This was proven by Reis *et al.* who coined the name LapB (*Listeria* adhesion protein B) and demonstrated *lapB*, which is absent from non-pathogenic *Listeria* species, to encode a sortase-dependent, cell wall-anchored adhesin required for entry into eukaryotic cells (37). Our observations and previous studies of others disclosed a regulation of *lapB* inverse to the one of LhrC1–5. Quillin *et al.* determined *lapB* to be down-regulated by more than 2-fold during bile stress (60), and 1 h of cefuroxime stress resulted in *lapB* down-regulation by a factor of about 2.5 (42). Both conditions cause strong induction of LhrC1–5 affirming our data that the sRNA negatively regulates *lapB* expression. In a comprehensive study on gene regulation in *L. monocytogenes*, all five copies of LhrC were found to be highly up-regulated in blood, and at the same time the amount of *lapB* mRNA was decreased (6). The adhesin was furthermore shown to be up-regulated in stationary phase and slightly down-regulated at 30°C. This correlates with results of the first report on LhrC in which it was described to be more highly expressed in exponential growth phase than in stationary phase and more prevalent under cold stress conditions (9).

LapB was demonstrated to play an important role for *L. monocytogenes’* virulence by mediating the adhesion to and invasion of non-phagocytic cells in a cell type-dependent manner (37). LhrC1–5 are possibly the critical regulators which ensure a coordinated expression of *lapB*, such as its repression in blood (6) to escape from the hosts immune response. Reis *et al.* assigned *lapB* to a whole group of genes that are down-regulated in blood but up-regulated in the spleen (37). This group of genes is composed of 11 others all associated with virulence or cell wall metabolism. It will be interesting to explore the assumption that more members of this group of genes represent targets of LhrC1–5 which could turn the sRNAs into an important regulatory hub controlling the proper site- and time-specific expression of several virulence factors during the process of listerial infection.

Although involvement of sRNAs in the regulatory network of virulence is beyond question (21,61), *lapB* represents one of the first virulence factors regulated by a trans-encoded sRNA in *L. monocytogenes*. To our knowledge only two further examples have been described so far. These are the expression of the listerial master regulator of virulence PrfA which is controlled by a trans-acting riboswitch (62), and the regulation of *chiA* mRNA, encoding for a virulence-associated chitinase (63), which is directly bound by the sRNA LhrA (26).

### Multiple copies of LhrC

Multicopy sRNAs have been reported for several bacteria (1,43), either being redundant (64), acting additively (65,66) or following a hierarchical mode of action (67,68). One of the few closer characterized examples in Gram-positive species is the five highly similar csRNAs (cia-dependent sRNAs) of *Streptococcus pneumonia* recently shown to be involved in competence regulation (69). Analogous to LhrC they are controlled by a TCS, CiaRH. For several of their targets an additive mode of action was proven. With respect to LhrC, β-gal assays revealed that all five *lhrC* promoters are active upon cell surface stress, showing the same induction pattern regardless of which kind of stress. This observation could lead to the assumption that the five LhrC copies act redundantly, i.e. one is capable of substituting for all others. However, a stress tolerance assay with cefuroxime and bile salts hampered not only the full *lhrC* mutant but also Δ*lhrC1–4* compared to wild type which rather indicates an additive action of the five copies. An additive mechanism can also be concluded from results of translational fusion experiments for the target *lapB*. The observed β-gal activity in the partial *lhrC* mutants Δ*lhrC1–4* and Δ*lhrC5* was measured to be in between the ones of the wild type and of the full *lhrC* mutant (Δ*lhrC1–5*). Although all five LhrC molecules are quite similar, they still show slight differences in their C-rich segments and flanking regions which could supply LhrC with a lot of flexibility in terms of target recognition as shown for the Qrr sRNAs in *V. harveyi* (66). Most likely, LhrC has more than only one target, and possibly its mechanism of action is different depending on the target. It is not always possible to distinctly categorize multicopy sRNAs as was exemplified in *Pseudomonas aeruginosa*. The sRNAs RsmY and RsmZ act redundantly in controlling type III secretion system, but an additive mechanism was found for the regulation of genes involved in biofilm formation (70). An identification of more LhrC targets and detailed studies on the single LhrC copies will certainly shed more light on the purpose of multiplicity and on how the five sRNA homologs interplay.

### A redundant CU-rich motif

LhrC is different from other multiple sRNAs described so far by featuring an additional dimension of multiplicity. The sRNA not only exists in five homologs, but each homolog exhibits three redundant CU-rich motifs that were shown to be actual sites of target recognition. It has been proposed that multiplicity of sRNAs permits a very sensitive reaction and helps to turn a small input signal into a large output response (1). Keeping five copies of LhrC with three internal copies of the regulatory motif, leads to the assumption, that these sRNAs control important adaptations that require ultra-sensitivity.

Repeated sequence elements in loops or single-stranded stretches of sRNA sites are reminiscent of protein-binding RNAs of the Rsm (repressor of secondary metabolites)/Csr (carbon storage regulator) family which have been discovered and characterized in several γ-proteobacteria (71). Originally, this elaborate mechanism of post-transcriptional regulation was discovered in *E. coli* (72). CsrA, a small protein which globally regulates gene expression by binding to mRNA targets can be sequestered by CsrB, a sRNA that hence antagonizes the effects of CsrA. Often several copies of the antagonizing sRNA exist. Some Pseudomonads exhibit three sRNAs (RsmZ, RsmY and RsmX) to antagonize the RNA binding proteins RsmA and RsmE, with RsmX existing in even five copies in *Pseudomonas syringae* (73). The fact that these sRNAs of Pseudomonads are all regulated by a TCS (GacAS) extends the observed analogy to listerial LhrC1–5. Even though the redundant motif of LhrC1–5 is different from the GGA motif that characterizes antagonizing sRNAs in γ-proteobacteria, one could speculate that LhrC1–5 could also function as protein-binding sRNAs, especially, since the dogma of an sRNA to be either protein-binding or RNA-binding was recently disproven by Jørgensen *et al.* who showed that McsA in *E. coli* not only directly binds to mRNAs but is also capable of capturing at least two copies of the global RNA-binding protein CsrA (74).

Strikingly, the redundant motif in LhrC comprises a sequence of UCCC. This C-rich motif is assumed to be a characteristic feature of sRNAs of low GC content bacteria that block translation initiation (51,75) by binding to the SD sequence. The involvement of the UCCC motif in mRNA target binding was proven for RNAIII (76), RsaE (51) and SprD (77) in *S. aureus* and for FsrA in *B. subtilis* (20). Indeed, also some of the sRNAs discovered in *L. monocytogenes* hold the UCCC motif in their structure (6,10). However, an involvement of the motif in target binding in *L. monocytogenes* is shown for the first time in this study.

The three sites containing the UCCC motif are located in both loops and in the single-stranded stretch between the two stem loops of the LhrC molecule. This appears plausible as loops and single stranded regions are known to be best suited for an initial contact of two RNA molecules (48–50). Besides the involvement of three locally separated sites of LhrC4 which all target the same specific site in *lapB*, LhrC4-*lapB* mRNA interaction holds another unexpected result as disclosed by structural probing experiments. Binding of LhrC4 to *lapB* mRNA entails a structural rearrangement in the sRNA, more specifically the 5′ site of stem A is engaged in a duplex with *lapB* mRNA extending the site of interaction into the coding region of the mRNA. Mutation of LhrC4 stem A did not have an effect on LhrC4-*lapB* mRNA binding efficiency in gel shift experiments. This observation is reminiscent of what has been described in the literature for RNA-OUT of IS10 transposition (48) and for FinP in the FinOP repressor system of plasmid R1 (78). In both cases mutations in a stem of an antisense RNA did not have an effect on target binding *in vitro*. As an explanation the authors argued that the stem was first involved in a secondary step of RNA-RNA interaction after the two RNAs had already made initial contact. One could speculate that the mechanism of LhrC4-*lapB* mRNA binding works in a similar way, i.e. the antisense RNA makes first contact with its RNA target in a very fast reaction restricted to only a few nucleotides most likely located in a loop structure (kissing), and subsequently this first complex is transferred into the ultimate RNA–RNA duplex in an irreversible and mostly not as rapid reaction (48,79). In the case of LhrC4, three sites could mediate first contact with *lapB* mRNA already inhibiting translational initiation (48–50). The irreversible step of final complex formation involving stem A can only occur starting from an initial interaction of *lapB* mRNA with loop A of LhrC4, as illustrated in Figure 7 and Supplementary Figure S15. Removal of this final RNA-RNA complex by RNases could work like a sink implicating that eventually all *lapB* molecules will take this path. In our study we were able to abolish binding of LhrC4 and *lapB* mRNA by mutating the three sites in LhrC4 capable of an interaction with *lapB* mRNA. A compensatory mutation of the *lapB* sequence could only partially restore interaction which we assume is caused by structural issues proven to be highly relevant for such RNA–RNA interactions (48–50,79). Future experiments with corresponding mutants *in vivo* have to be performed to corroborate the suggested interactions between LhrC and *lapB* mRNA.

A plethora of non-coding sRNAs in scores of bacterial species have been identified throughout the last decade. With respect to pathogenic bacteria one hopes to exploit these regulatory sRNAs in anti-microbial strategies one day. However, there is still a long way to go since most of them are completely uncharacterized. This study unveiled details on the sRNAs LhrC1–5 concerning conditional and regulatory requirements for their expression, presents the mRNA of a virulence-associated adhesin as their first direct target, and shows interesting new mechanistic aspects on sRNA-mRNA interaction. The association with *L. monocytogenes* virulence and the sophisticated way of target binding calls for further in-depth analyses on these five homologous sRNAs.

## SUPPLEMENTARY DATA

Supplementary Data are available at NAR Online.

SUPPLEMENTARY DATA
