# Supplementary material for: A multicopy sRNA of Listeria monocytogenes regulates expression of the virulence adhesin LapB
Source: Nucleic Acids Res. 2014 Jul 17;42(14):9383–98. doi: 10.1093/nar/gku630 (PMC4132741; doi:10.1093/nar/gku630)
Supplement: SUPPLEMENTARY DATA [file supp_42_14_9383__index.html]

A multicopy sRNA of Listeria monocytogenes regulates expression of the virulence adhesin LapB — SUPPLEMENTARY DATA 

# A multicopy sRNA of *Listeria monocytogenes* regulates expression of the virulence adhesin LapB

## SUPPLEMENTARY DATA

**Files in this Data Supplement:**

- SUPPLEMENTARY DATA
