# Supplementary material for: A multicopy sRNA of Listeria monocytogenes regulates expression of the virulence adhesin LapB
Source: Nucleic Acids Res. 2014 Jul 17;42(14):9383–98. doi: 10.1093/nar/gku630 (PMC4132741; doi:10.1093/nar/gku630)
Supplement: SUPPLEMENTARY DATA [file supp_gku630_nar-00725-v-2014-File009.pdf]

A multicopy sRNA of *Listeria monocytogenes* regulates expression of the virulence adhesin LapB

Susanne Sievers, Eva Maria Sternkopf Lillebæk, Kirstine Jacobsen, Anja Lund, Maria Storm Mollerup, Pia Kiil Nielsen and Birgitte Haahr Kallipolitis*

Department of Biochemistry and Molecular Biology, University of Southern Denmark, Odense, Denmark

*corresponding author: Department of Biochemistry and Molecular Biology, University of Southern Denmark, Odense, Denmark. Tel.: +45 6550 2372. E-mail: bhk@bmb.sdu.dk

Supplementary Data

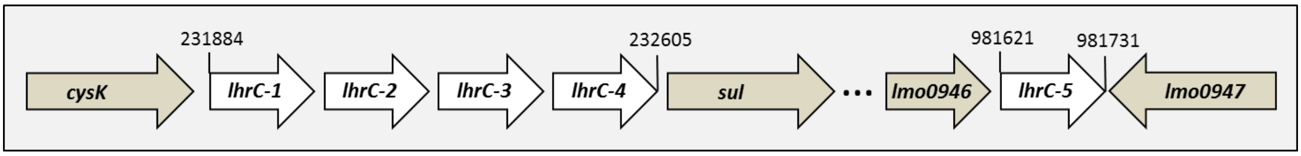

Figure S1. Schematic location of *lhrC*. All five copies of *lhrC* are encoded on the positive DNA strand, *lhrC1* to *lhrC4* clustered, but *lhrC5* at a distant site. The gene map is according to Genolist (1). Scaling and distances do not reflect reality. An overview of the genetic organization of *lhrC* in various *L. monocytogenes* serotypes and non-pathogenic *Listeria* species was provided by Mraheil *et al.* (2).

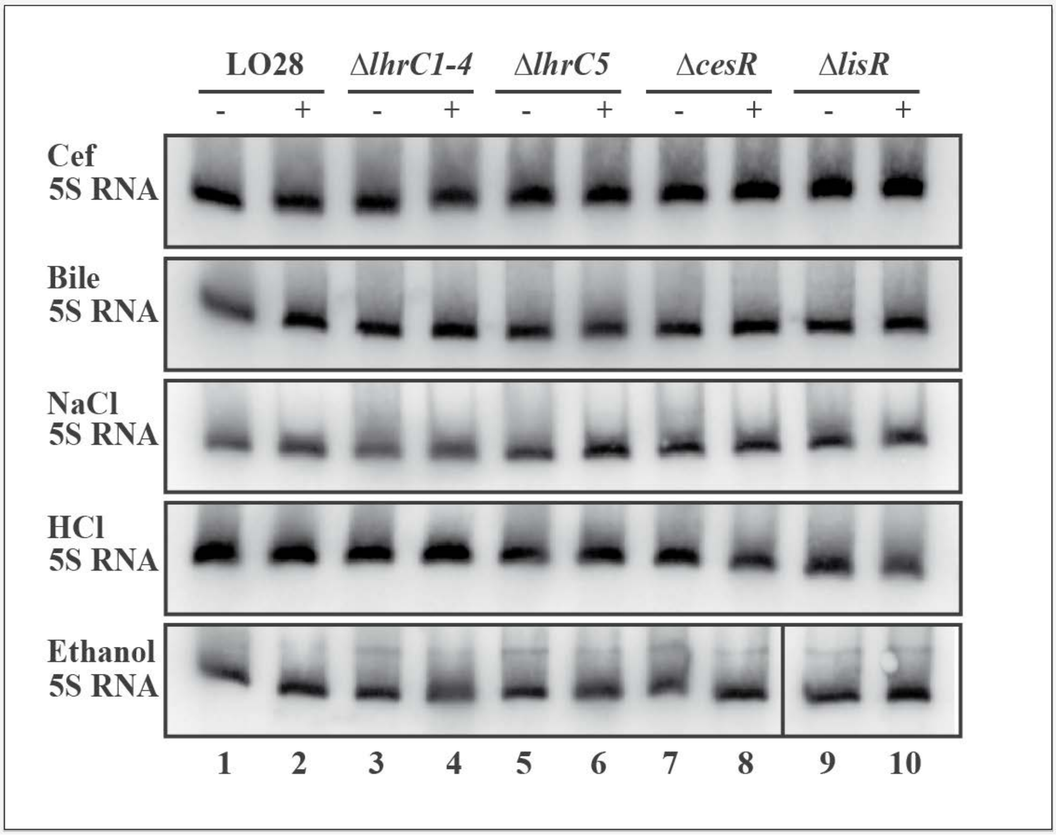

Figure S2A. Northen blot probed for 5S rRNA. Loading control for northen blot analysis of LhrC shown in Figure 1A. Samples were taken from *L. monocytogenes* LO28 wild type (lane 1, 2), Δ*lhrC1-4* (lane 3, 4), Δ*lhrC5* (lane 5, 6), Δ*cesR* (lane 7, 8) and Δ*lisR* (lane 9, 10) from cultures stressed with various agents acting on the cell envelope (+) as well as from control samples without stress (-).

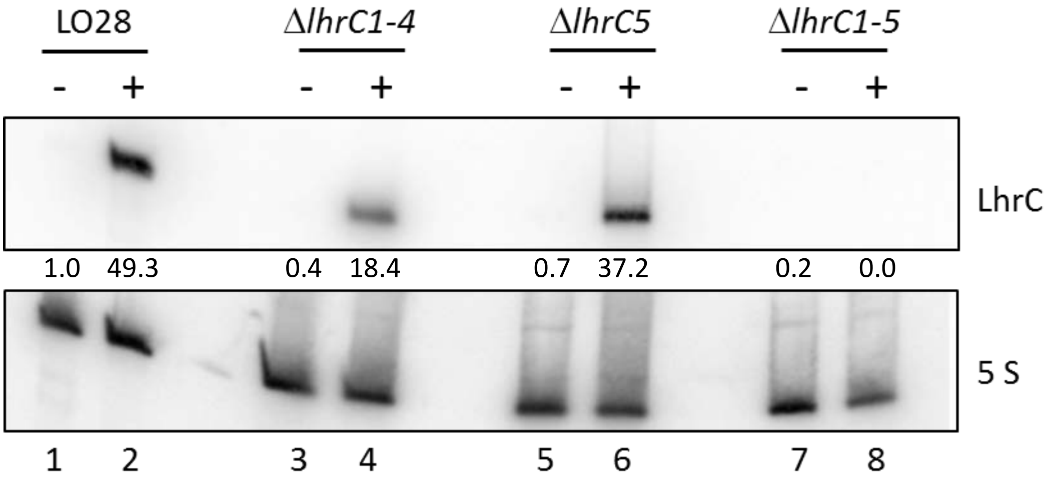

Figure S2B. Absence of LhrC in Δ*lhrC1-5* checked by Northern blot analysis. Samples were taken from *L. monocytogenes* LO28 wild type (lanes 1 and 2), Δ*lhrC1-4* (lanes 3 and 4), Δ*lhrC5* (lanes 5 and 6) and Δ*lhrC1-5 (*lanes 7 and 8) from cultures stressed with 4 µg/mL cefuroxime (+) as well as from control cultures without stress (-). The Northern blot was probed for LhrC1-5 and 5S rRNA (loading control). The levels of LhrC were normalized to 5 S.

A

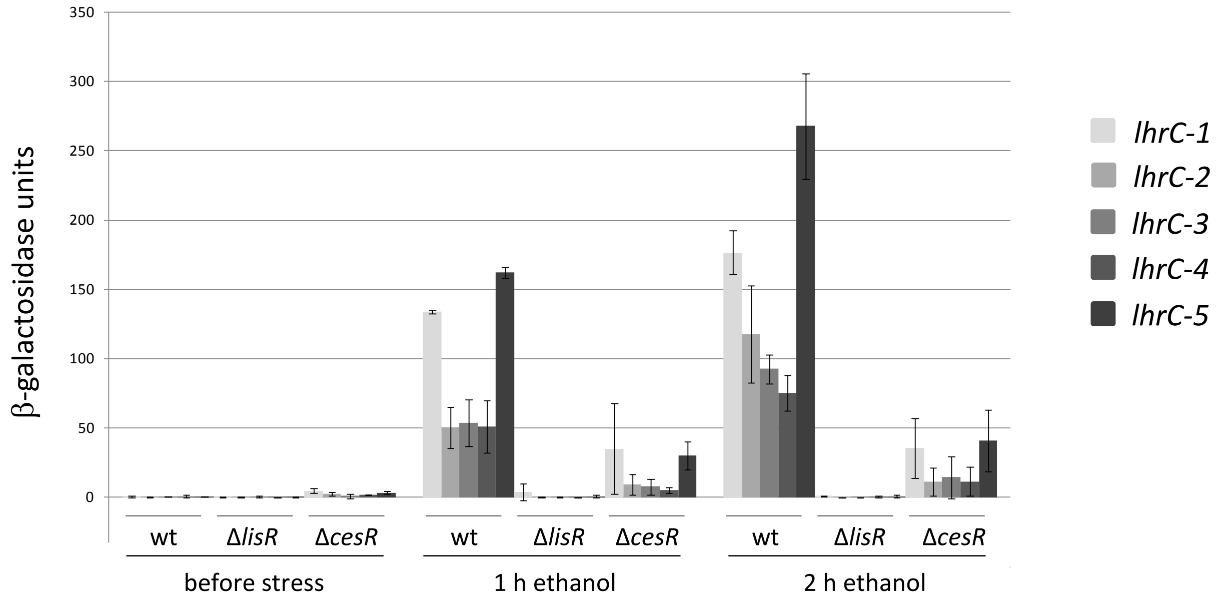

B

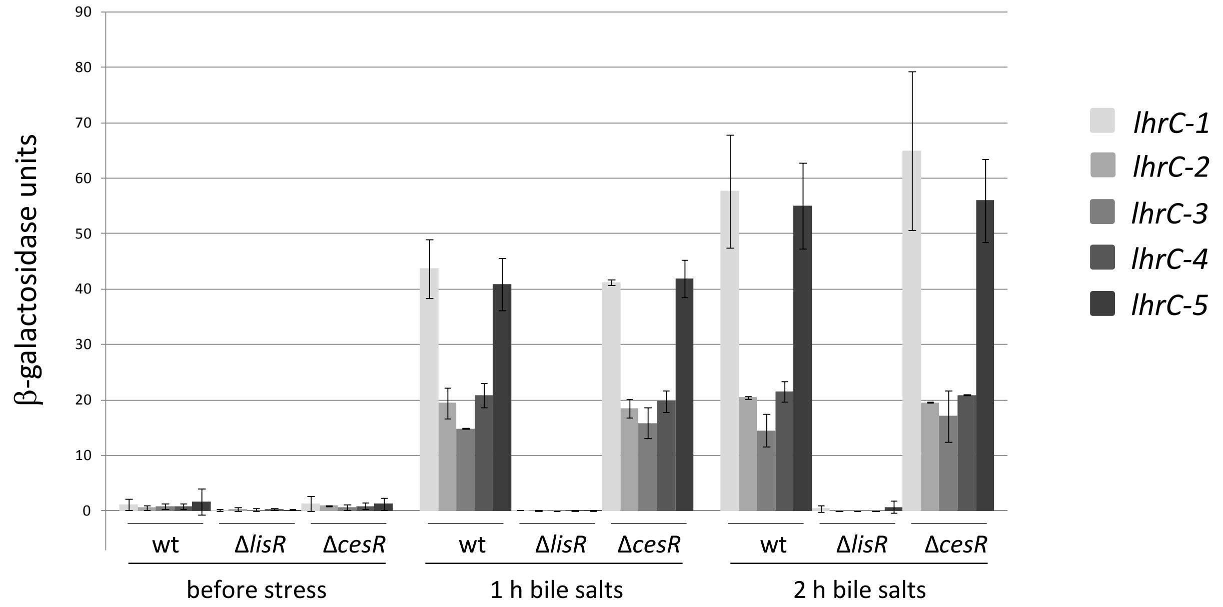

Figure S3. Transcriptional reporter gene fusions of *lhrC* promoters. Reporter fusions were tested in LO28 wild type (wt), Δ*lisR* and Δ*cesR* strains. Cultures were grown up to OD_{600}=0.35 and stressed with ethanol (A) and bile salts (B), after control samples had been taken. Further samples for β-gal assays were withdrawn after 1 and 2 h of ongoing stress. Results represent the average of two biologically independent experiments, each in technical duplicates.

A

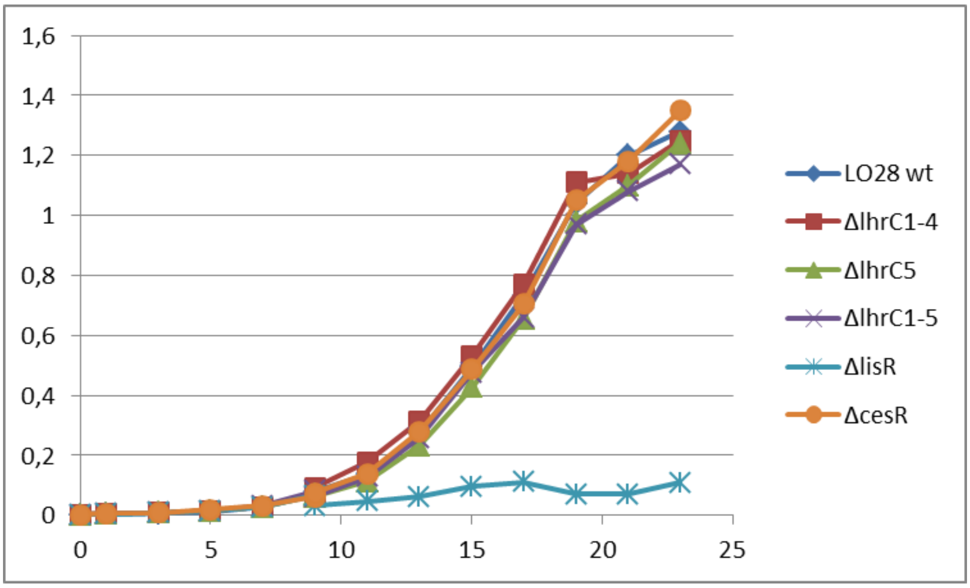
1,6
1,4
1,2
1
0,8
0,6
0,4
0,2
0
0
5
10
15
20
25
LO28 wt
ΔlhrC1-4
ΔlhrC5
ΔlhrC1-5
ΔlisR
ΔcesR

B

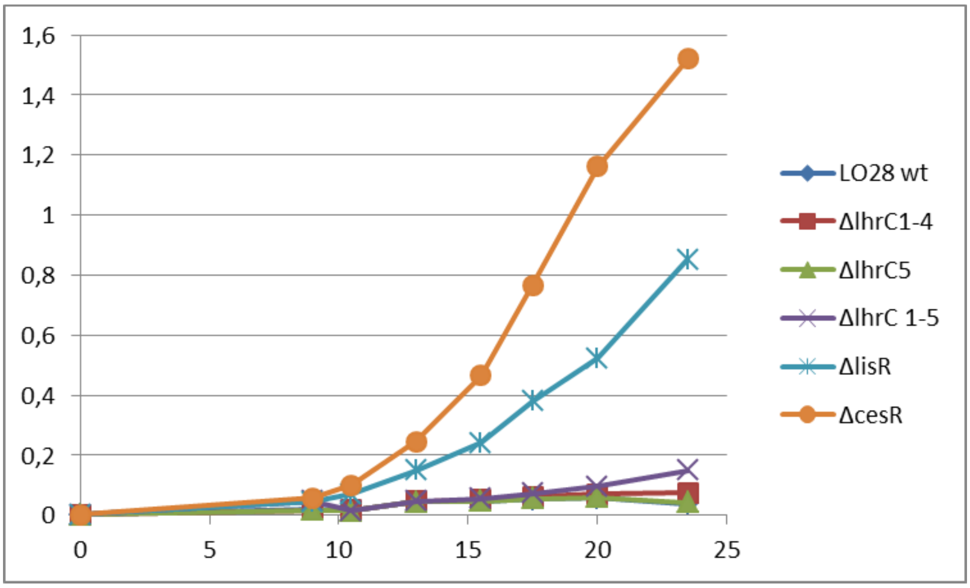
1,6
1,4
1,2
1
0,8
0,6
0,4
0,2
0
0
5
10
15
20
25
LO28 wt
ΔlhrC1-4
ΔlhrC5
ΔlhrC 1-5
ΔlisR
ΔcesR

C

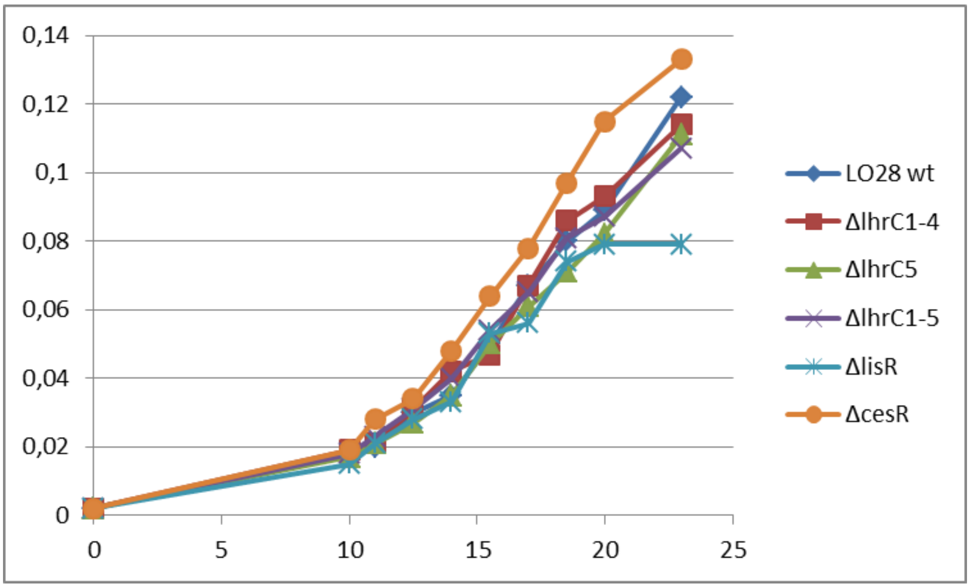

D

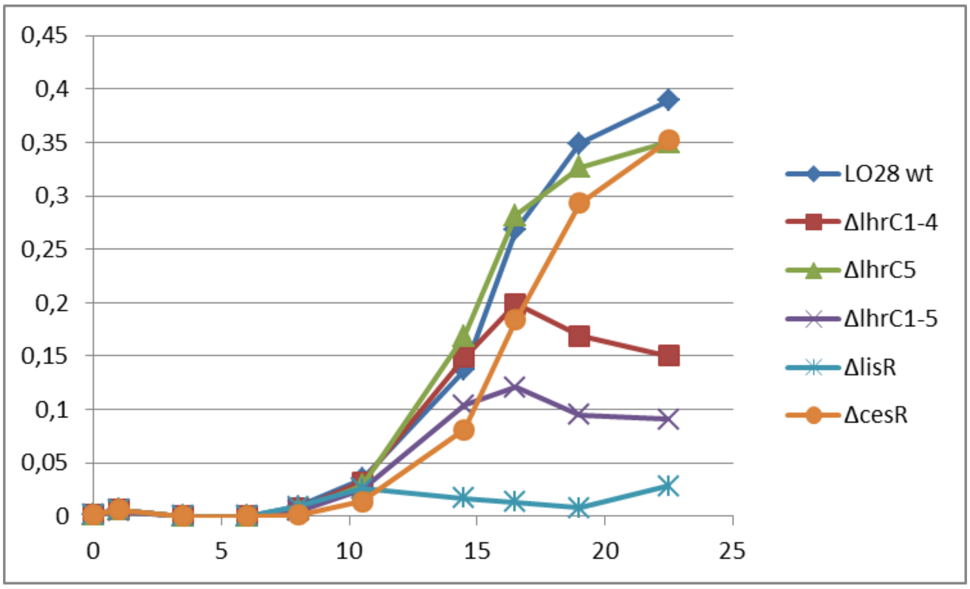

Figure S4. Stress tolerance assays. Overnight cultures were diluted 1000-fold into BHI adjusted with (A) acid (pH 5), (B) ethanol (5%), (C) NaCl (8%) or (D) bile salts (0.07%). Growth was monitored up to 24 hours. The result from one representative growth experiment is shown. Each experiment was repeated twice with similar results.

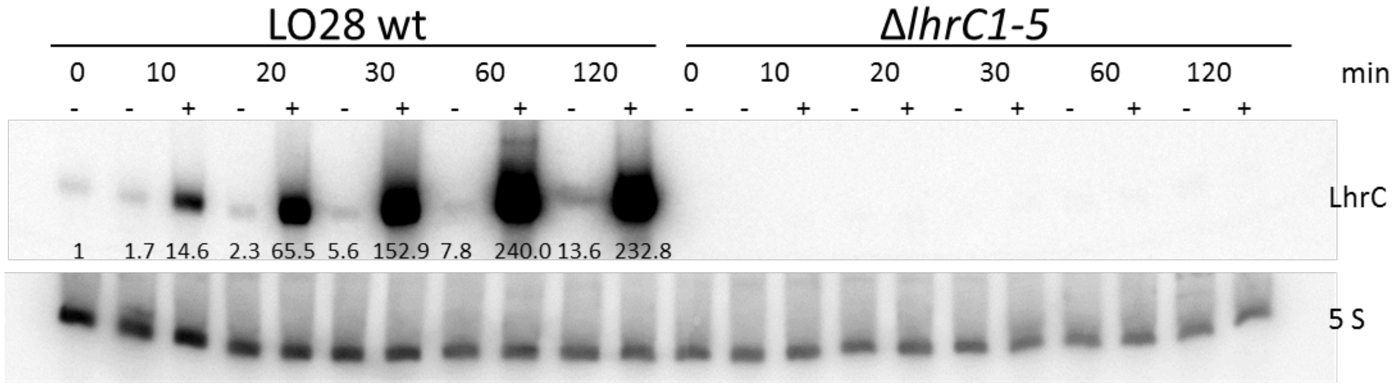

Figure S5. LhrC induction profile. At time = 0, corresponding to OD_{600} = 0.35, *L. monocytogenes* LO28 wild type and Δ*lhrC1-5* cells were treated with 4 µg/ml cefuroxime. Samples were harvested at several time points (0, 10, 20, 30, 60 and 120 min relative to the addition of cefuroxime) and total RNA was prepared for northern blot analyses, probing for LhrC1-5 as well as 5S rRNA (loading control). Levels of LhrC were normalized to 5S. No LhrC was detected in the Δ*lhrC1-5* strain. In the wild type strain the highest amount of LhrC was observed after 1 h of stress. Also the strongest fold of induction of LhrC compared to the non-stress condition was taking place 1 h after stress was set (30 fold).

A

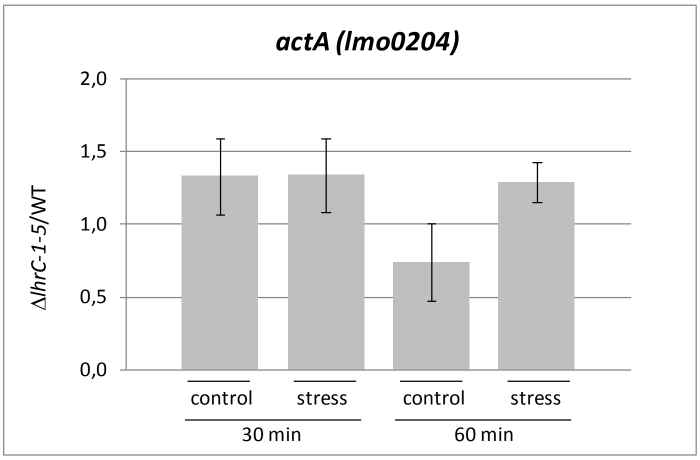

B

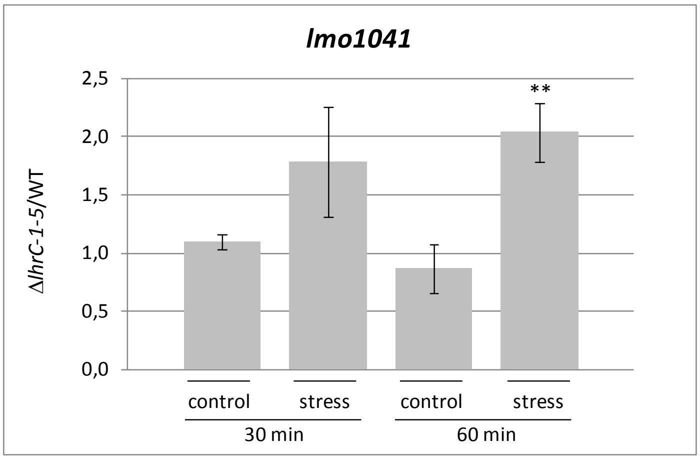

C

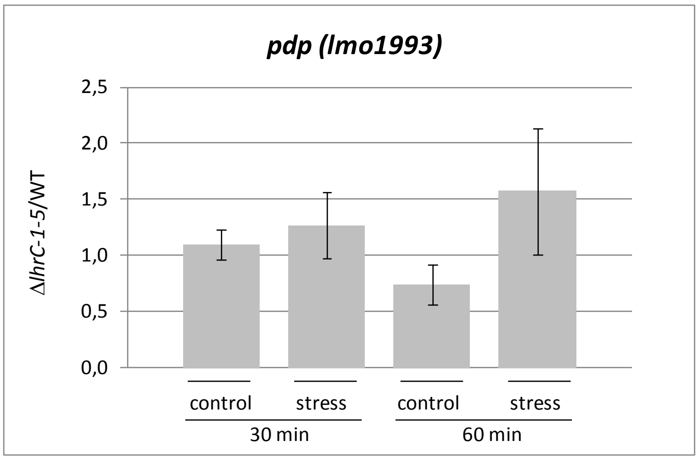

Figure S6A. RT-qPCR analysis of *actA* (A), *lmo1041* (B) and *pdp* (C). At OD_{600} = 0.35, wild type and Δ*lhrC1-5* cultures were split and half of the cultures were treated with 4 µg/mL cefuroxime (stress) whereas the other half was left untreated (control). Samples were harvested 30 and 60 min after the addition of cefuroxime and total RNA was prepared for qRT-PCR analysis in three independent experiments. Two asterisks indicate a significant increase of the ratio under stress conditions compared to the corresponding control, with $P < 0.005$.

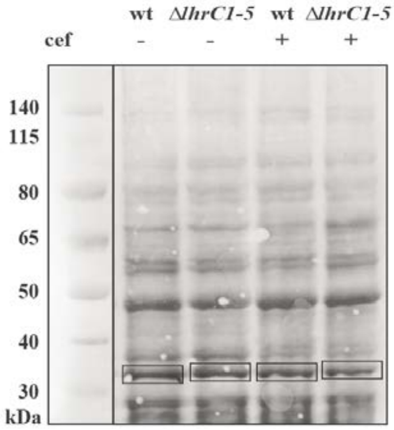

Figure S6B. Amido black stained western blot membrane. Loading control for western blot analysis of LapB shown in Figure 3B. Samples were taken 2 h post treatment with cefuroxime (4 µg/ml) from wild type (wt) and Δ*lhrC1-5*, but also from unstressed cultures. Surface protein enriched extracts were separated via 1D-PAGE. Bands quantified for normalization of LapB levels are boxed.

−35 −10

5´-**cttgcttttttcaagaacaatagtaaaataagttata**tcaagtttgtcatagataaaataggg
gaataacatgacaatagctttaggaatggggataaaataattggggacttttaaaaaagggagat
ggggatgaatgaaaagtaaattttttataggcatgatgac — *lacZ*

Figure S7. The pC-*lapB-lacZ* construct. The core promoter region of the *lhrA* gene (bold) was fused to a fragment extending from -100 to +32 relative to the *lapB* translational start site (atg is underlined). The resulting fragment was fused in frame to *lacZ* in the translational fusion vector pCK-lac (3).

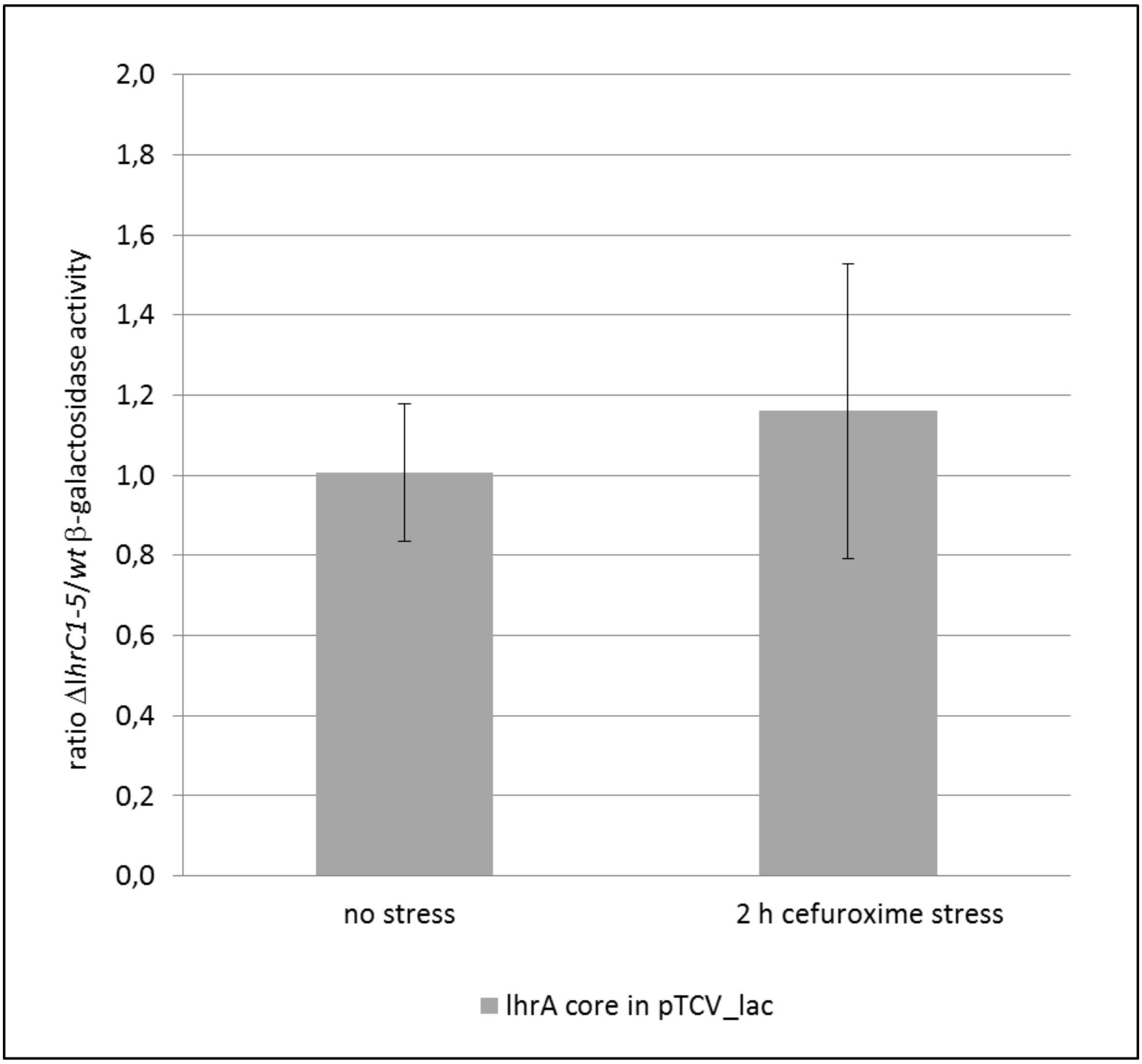

Figure S8. The core promoter is not affected by LhrC. The *lhrA* core promoter was fused to *lacZ* in the transcriptional fusion vector pTCV-lac (4). Transcriptional regulation of the core promoter in wild type and Δ*lhrC1-5* was similar under control conditions as well as after cefuroxime stress. Results are the average of two biologically independent experiments, each in technical duplicates.

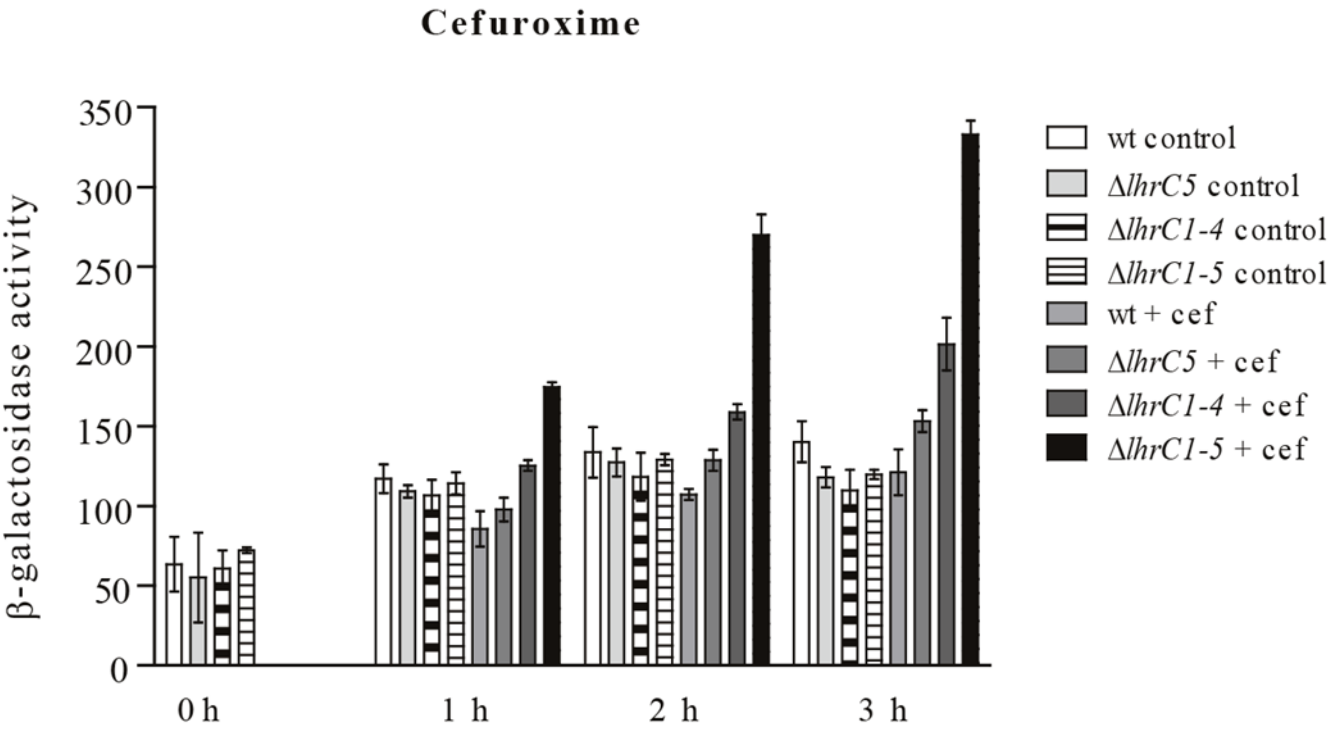

Figure S9. Testing the effect of LhrC1-4 and LhrC5 on *lapB-lacZ* expression. β-galactosidase assay of pC-*lapB-lacZ* in strains lacking LhrC1-4 or LhrC5 was compared to LO28 wild type and the mutant strain lacking all five copies of LhrC. Cells were grown to OD_{600} = 0.2 and cells were sampled (0 h) before the cultures were split. One sub-culture was treated with cefuroxime (cef) to a final concentration of 4 µg/ml whereas the other one was left untreated (control). Samples were taken at 1, 2 and 3 hours relative to the onset of cefuroxime exposure. Results are the average of three biological experiments, each performed in duplicate.

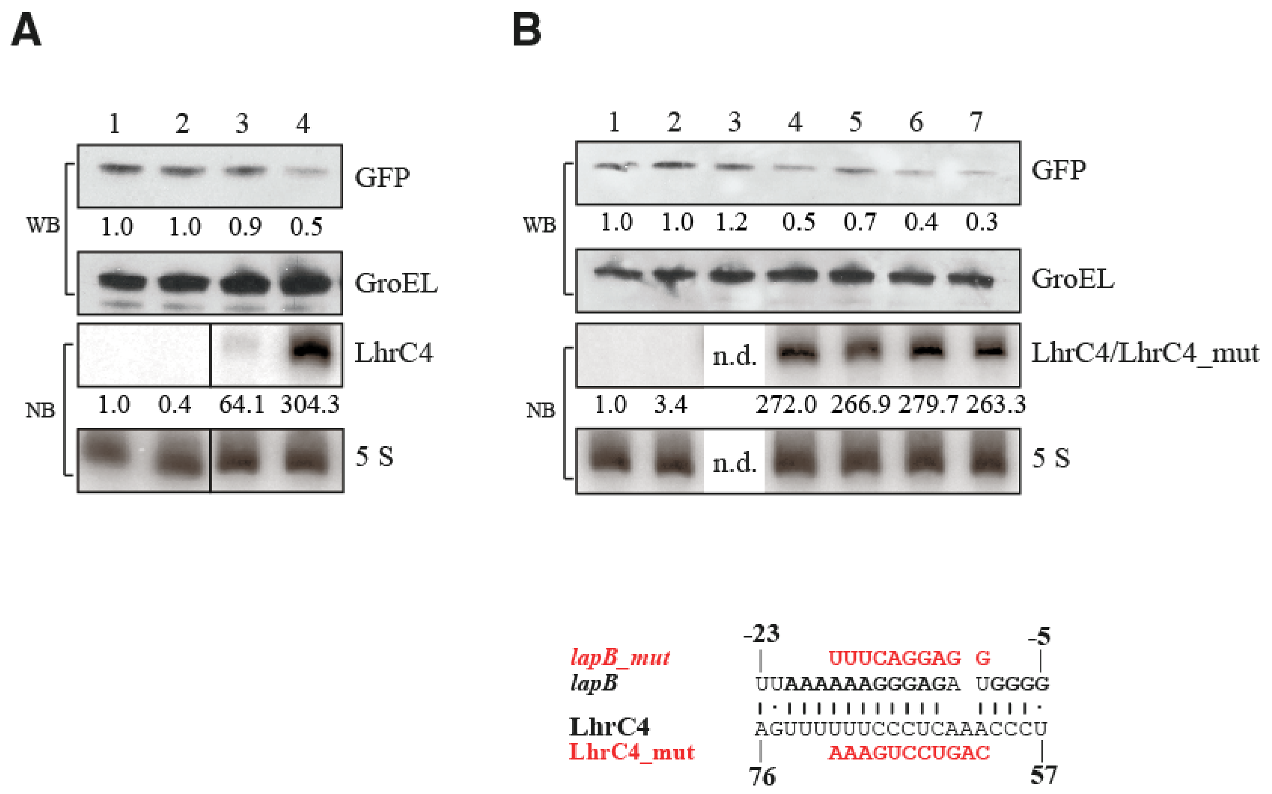

Figure S10. Simultaneous overexpression of sRNA and target RNA in *E. coli*. (A) *lapB* fused to a GFP reporter gene was constitutively expressed from pXG-10_*lapB* in *E. coli* (lanes 1-4). *E. coli* harboring pXG-10_*lapB* and the empty vector pNDM220, was grown in the absence (lane 1) or presence (lane 2) of IPTG. *E. coli* carrying pXG-10_*lapB* and pNDM220_*lhrC4*, was grown in the absence (lane 3) or presence (lane 4) of IPTG. The GFP signal on a western blot (WB) was diminished when LhrC4 expression was induced with IPTG from pNDM220 (lane 4). LhrC4 expression was verified by northern blot (NB) analysis. Relative levels of GFP and LhrC (normalized to controls) are shown between the bands. **(B)** The *E. coli* strain harbored the following plasmids: pXG-10_*lapB* + pNDM220 (lane 1); pXG-10_*mut_lapB* + pNDM220 (lane 2); pXG-10_*lapB* + pNDM220_*rprA* (lane 3); pXG-10_*lapB* + pNDM220_*lhrC4* (lane 4); pXG-10_*mut_lapB* + pNDM220_*lhrC4* (lane 5); pXG-10_*lapB* + pNDM220_*mut_lhrC4* (lane 6); or pXG-10_*mut_lapB* + pNDM220_*mut_lhrC* (lane 7). The strains were grown in the presence of IPTG. The mere mutation of the *lapB* sequence (in the absence of LhrC4) did not change GFP expression level (lane 2). Expression of LhrC4 simultaneous with *lapB* resulted in a decrease of GFP signal intensity (lane 4) whereas expression of an unspecific sRNA (RprA, lane 3) did not. The mutated *lapB* sequence interacted less well with native LhrC4, reflected by an increase in the GFP signal (lane 5). Curiously, a corresponding LhrC4 mutant was still capable of binding to wild type *lapB* (lane 6) as well as the mutated *lapB* (lane 7). A comparable expression level of LhrC4 and mutated LhrC4 was ensured by detection of the sRNAs on a northern blot (NB). N.d.: Not determined. In the illustration of the predicted LhrC4-*lapB* mRNA interaction, the mutations in *lapB* and LhrC4 are shown in red. Relative levels of GFP and LhrC (normalized to controls) are shown between the bands.

1	2	3	4	5	6	7	8	9	10	11	12	Sample
-	-	-	-	-	-	+	+	+	+	+	+	10 µM Hfq
500	0	4	20	100	500	500	0	4	20	100	500	nM *lapB*
125	-		5	25	125	125	-		5	25	125	Fold excess *lapB*
0.28	1.00	0.96	0.93	0.34	0.13	0.20	1.00	1.00	0.57	0.22	0.06	Unbound LhrC

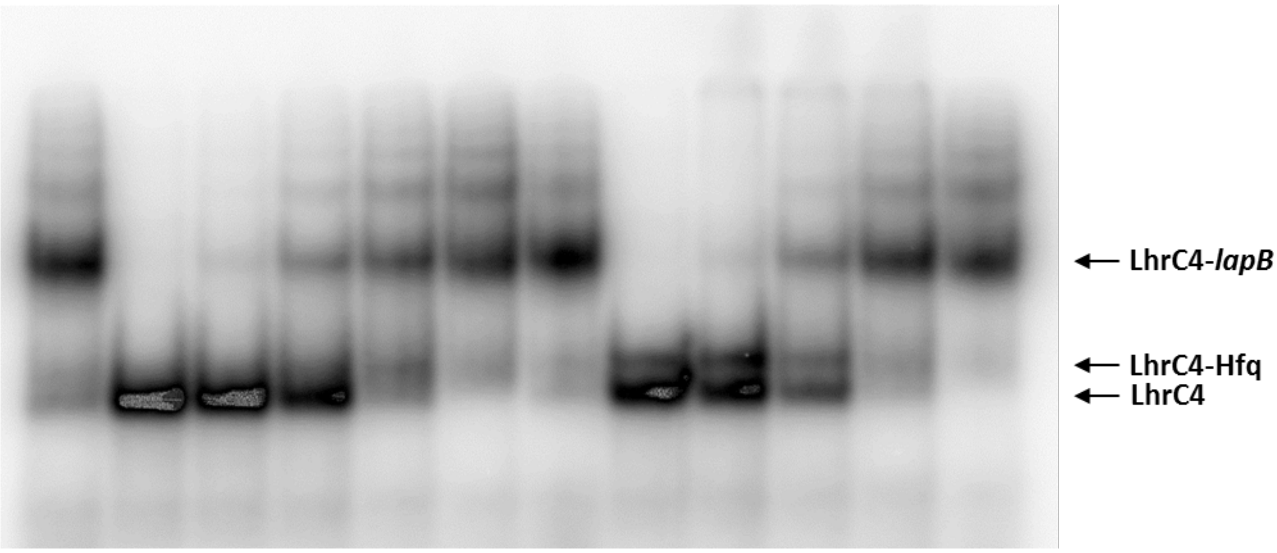

Figure S11. Role of Hfq in LhrC4-*lapB* binding. Labelled LhrC4 was shifted with increasing amounts of *lapB*, in the absence (lanes 1 to 6) or presence of 10 µg/ml Hfq (lanes 7 to 12). "Fold excess *lapB*" refer to the amount of lapB RNA added to each sample, relative to the amount of labelled LhrC. In lanes 1 and 7, RNAs were heat-denatured before allowing them to interact to form the most stable complex. The level of LhrC not bound to *lapB* is show ("unbound LhrC").

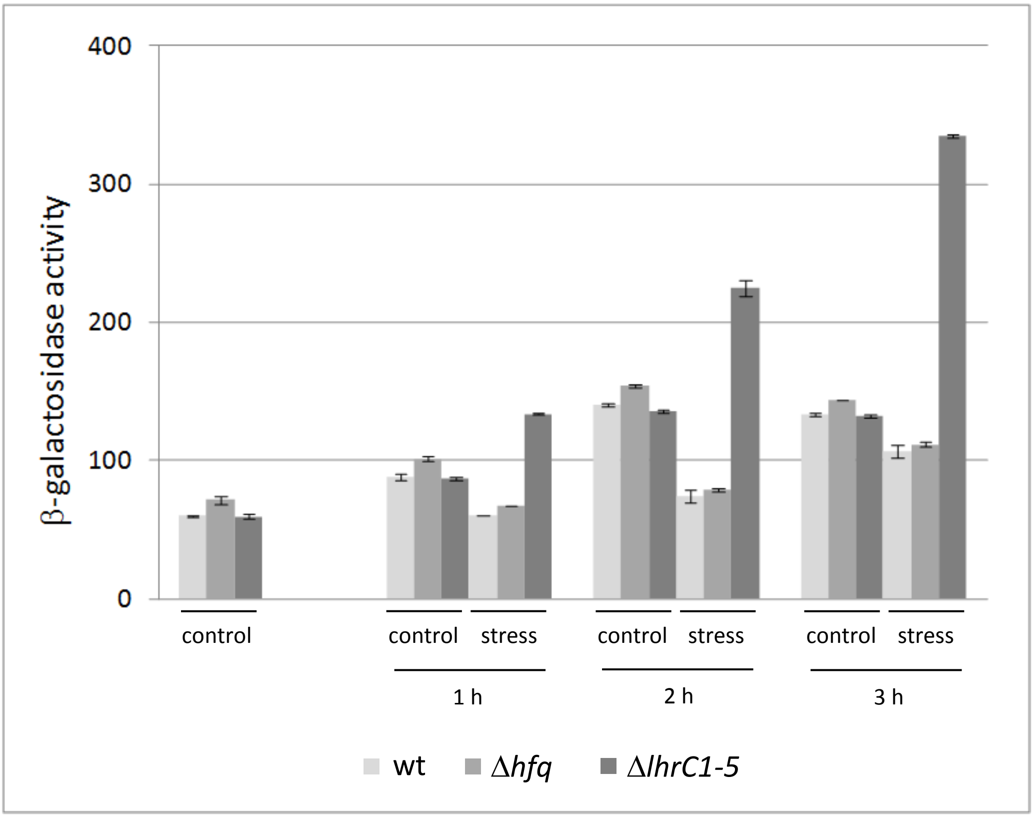

Figure S12. pC-*lapB-lacZ* in a Δ*hfq* background. LO28 wild type, Δ*hfq* and Δ*lhrC1-5* strains carrying p*C-lapB-lacZ* were grown to OD_{600} = 0.2 (control). The culture was split in two and 4 µg/ml cefuroxime was added to one of the cultures. After 1, 2 and 3 hours of growth, samples were taken from the unstressed cultures (control) and cultures exposed to cefuroxime (stress). β-galactosidase activity in Δ*hfq* was comparable to wild type, but not to Δ*lhrC1-5* background, where it was significantly increased after cefuroxime stress. Hence, LhrC is capable of regulating expression from pC-*lapB-lacZ* despite the absence of Hfq. Results are the average of two biologically independent experiments, each in technical duplicates.

A

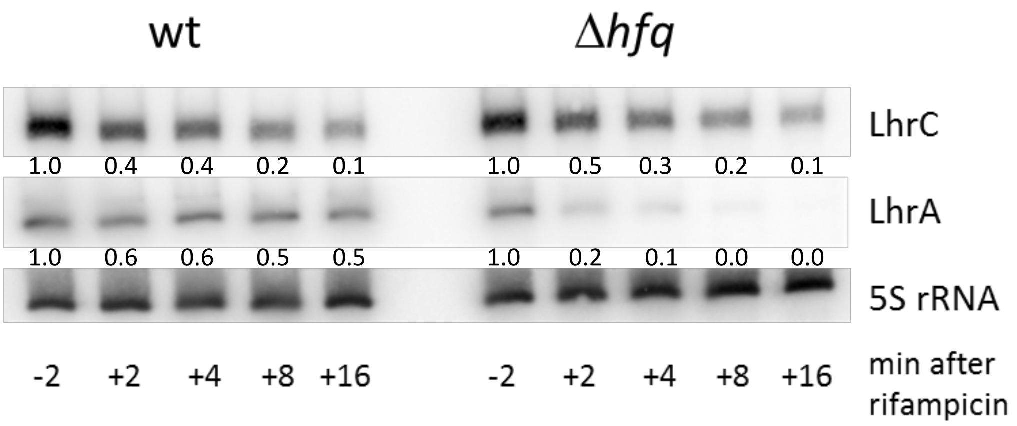
wt
Δhfq
LhrC
1.0 0.4 0.4 0.2 0.1 1.0 0.5 0.3 0.2 0.1
LhrA
1.0 0.6 0.6 0.5 0.5 1.0 0.2 0.1 0.0 0.0
5S rRNA
-2 +2 +4 +8 +16 -2 +2 +4 +8 +16
min after rifampicin

B

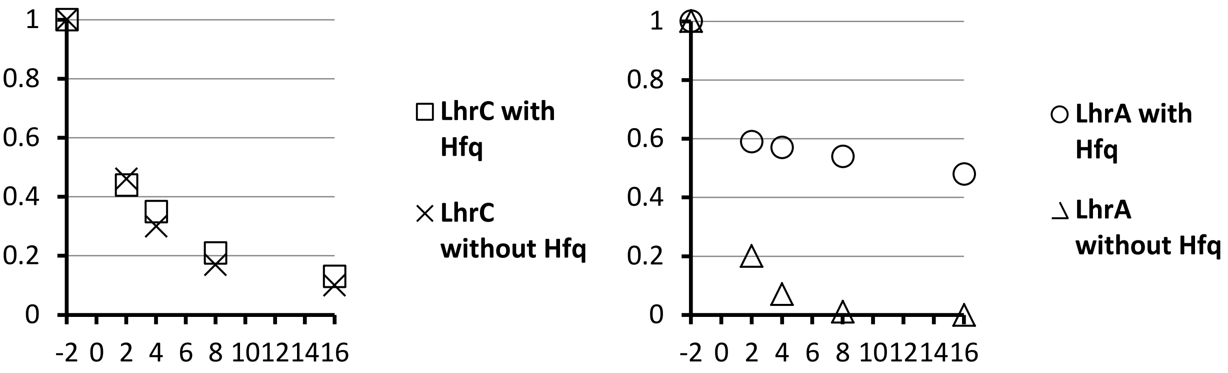
1
0.8
0.6
0.4
0.2
0
-2 0 2 4 6 8 10 12 14 16
LhrC with Hfq
LhrC without Hfq
LhrA with Hfq
LhrA without Hfq

C

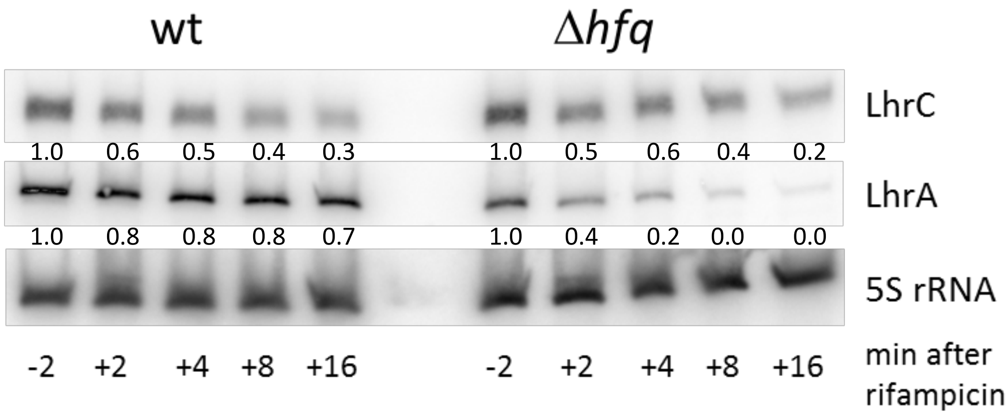

D

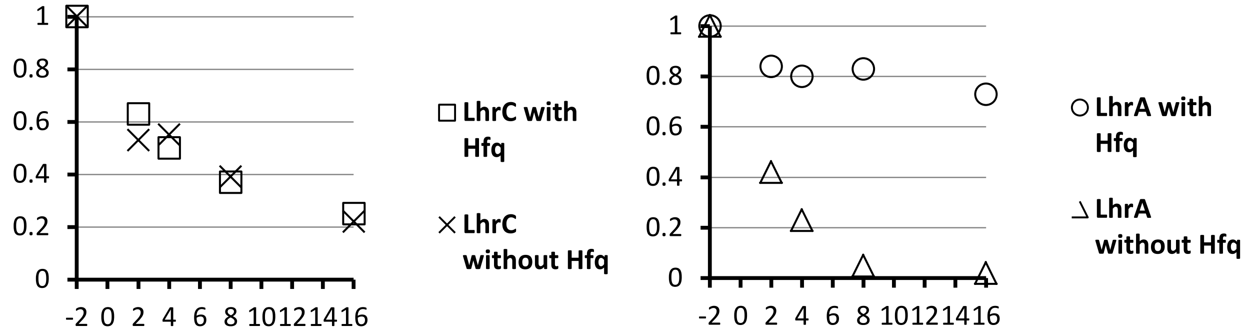

Figure S13. The effect of Hfq on LhrC stability. RNA was extracted from *L. monocytogenes* LO28 wild type and Δ*hfq* cells stressed with cefuroxime (A) or bile salts (C) for 30 min before transcription was inhibited by the addition of rifampicin (10 µg/ml). Samples were taken shortly before rifampicin addition (-2 min) and in a time course afterwards. As a positive control, LhrA, a sRNA whose stability was earlier reported to be dependent on Hfq was probed for (5). 5S rRNA is shown as a loading control. The levels of LhrC and LhrA (normalized to 5S) are shown below the bands. A graphical representation of normalized LhrC and LhrA levels are shown in (B) for stress with cefuroxime and (D) for bile salts. The estimated half-life for LhrC subjected to either cefuroxime or bile salt stress is 2-3 min, independent of the presence or absence of Hfq. For LhrA in the wild type strain, the half-life is equal to or longer than 16 min for cefuroxime and bile stress, respectively, but in the absence of Hfq, the half-life is greatly reduced, estimated to less than 2 min. All three RNAs were detected on the same northern blot with intermediate membrane stripping.

LhrC
AUAAGCUAACAACAAACAAAACAUUUUCAUUCUUCUCCCCCCUUUUAGAAUGAAAAUCCCAAACUCCCUUUUUUGACCGA
UGCGGUUUACUCCCUUUUCCGCAUCGGUUUUUUU

LhrC_mut_2
AUAAGCUAACAACAAACAAAACAUUUUCAUUCUUCUCCCCCCUUUUAGAAUGAAAAUAGAACAGAAGGAACCCCGACCGA
UGCGGUUUACUCCCUUUUCCGCAUCGGUUUUUUU

LhrC_mut_3
AUAAGCUAACAACAAACAAAACAUUUUCAUUCUUCUCCCCCCUUUUAGAAUGAAAAUCCCAAACUCCCUUUUUUGACCGA
UGCGGACUAGAGGGAAAACCGCAUCGGUUUUUUU

LhrC_mut_4
AUAAGCUAACAACAAACAAAACAUUUUCAUUCUAAUGGCGGGAAAAAGAAUGAAAAUCCCAAACUCCCUUUUUUGACCGA
UGCGGUUUACUCCCUUUUCCGCAUCGGUUUUUUU

LhrC_mut_5
AUAAGCUAACAACAAACAAAACAUUUUCAUUCUUCUCCCCCCUUUUAGAAUGAAAAUAGAACAGAAGGAACCCCGACCGA
UGCGGACUAGAGGGAAAACCGCAUCGGUUUUUUU

LhrC_mut_6
AUAAGCUAACAACAAACAAAACAUUUUCAUUCUAAUGGCGGGAAAAAGAAUGAAAAUAGAACAGAAGGAACCCCGACCGA
UGCGGUUUACUCCCUUUUCCGCAUCGGUUUUUUU

LhrC_mut_7
AUAAGCUAACAACAAACAAAACAUUUUCAUUCUAAUGGCGGGAAAAAGAAUGAAAAUAGAACAGAAGGAACCCCGACCGA
UGCGGACUAGAGGGAAAACCGCAUCGGUUUUUUU

LhrC_mut_8
AUAAGCUAACAACAAACAAAACAUUUUCAUUCUAAUGGCGGGAAAAAGAAUGAAAAUCCCAAACUCCCUUUUUUGACCGA
UGCGGACUAGAGGGAAAACCGCAUCGGUUUUUUU

Figure S14. Overview of substitutions in LhrC_mut_2 through LhrC_mut_8. Shown are the sequences of LhrC wild type and mutant derivatives tested for their ability to bind *lapB* mRNA in the experiment presented in Figure 5. The mutated regions are underlined.

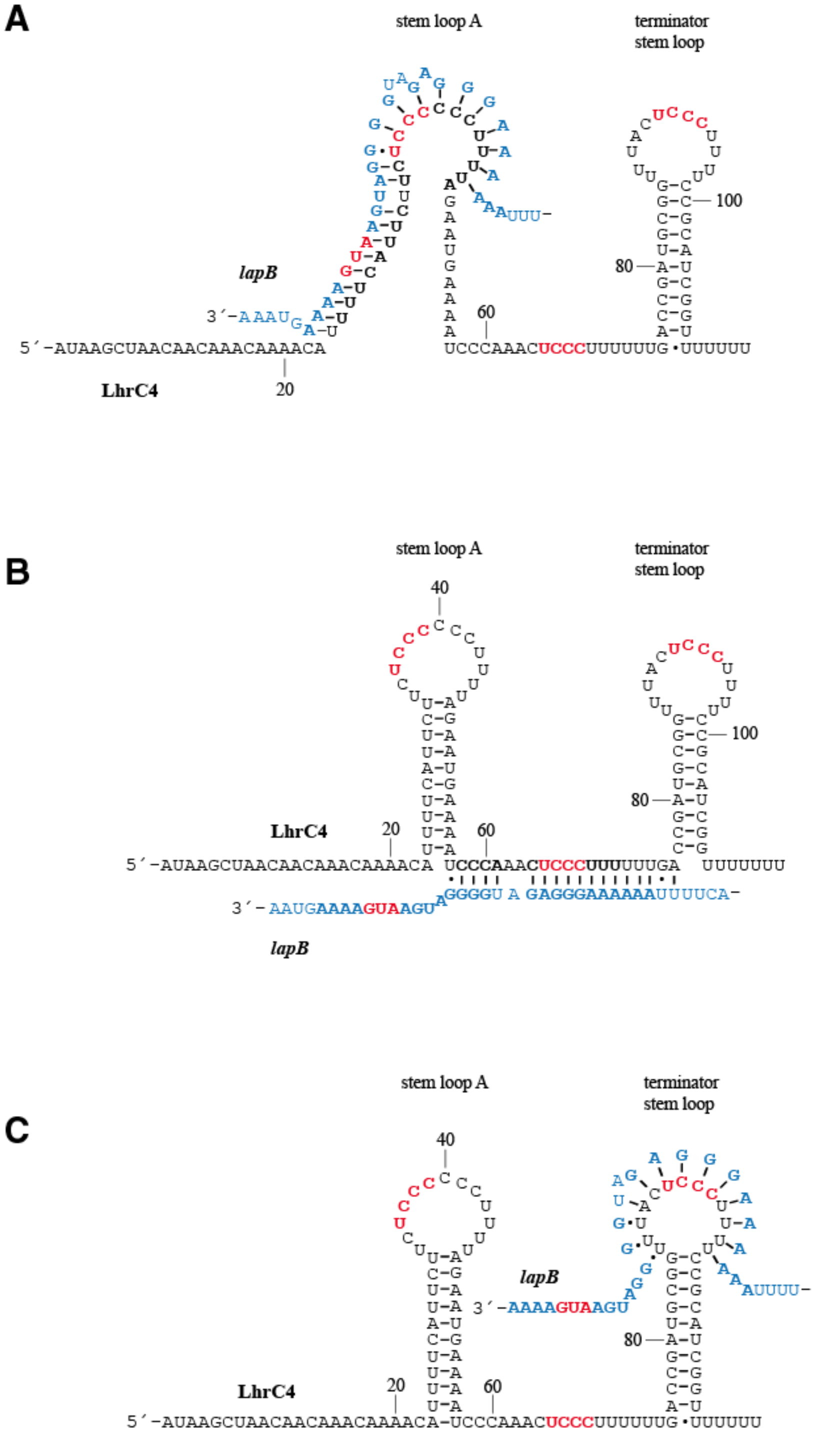

Figure S15. Detailed model of LhrC4-*lapB* mRNA interaction. Deduced base pairing of *lapB* mRNA and loop A (A), the single stranded stretch (B) and the terminator loop (C) of LhrC4, respectively. The *lapB* mRNA is shown in blue and the start codon is marked in red. The *lapB* mRNA and LhrC4 sequence found to be bound in structure probing experiments is printed in bold. The UCCC motif in LhrC4 is shown in red.

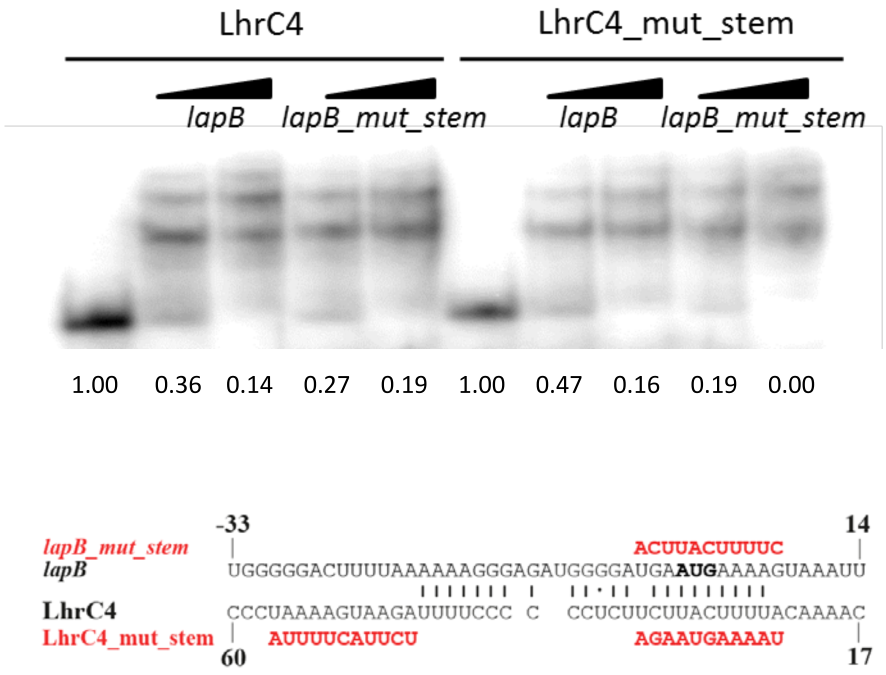

Figure S16. Role of stem A in LhrC4-*lapB* interaction. Labelled LhrC4 (lanes 1-5) or LhrC4_mut_stem (lanes 6-10) were shifted with increasing concentrations of *lapB* (lanes 2-3 and lanes 7-8) or *lapB_mut_stem* (lanes 4-5 and lanes 9-10). In lanes 2, 4, 7 and 9, 25 fold excess of *lapB/lapB_mut_stem* was used; in lanes 3, 5, 8, and 10, we used 125 excess of *lapB/lapB_mut_stem*. Inversion of the sequence of stem A (LhrC4_mut_stem) did not hamper binding to *lapB*, and *lapB* holding a compensatory mutation to LhrC4_mut_stem could still interact with LhrC4. In the illustration of the predicted LhrC4-*lapB* mRNA interaction, the mutations in *lapB* and LhrC4 are shown in red. Levels of unbound LhrC4/LhrC4_mut_stem are indicated below the gelshift.

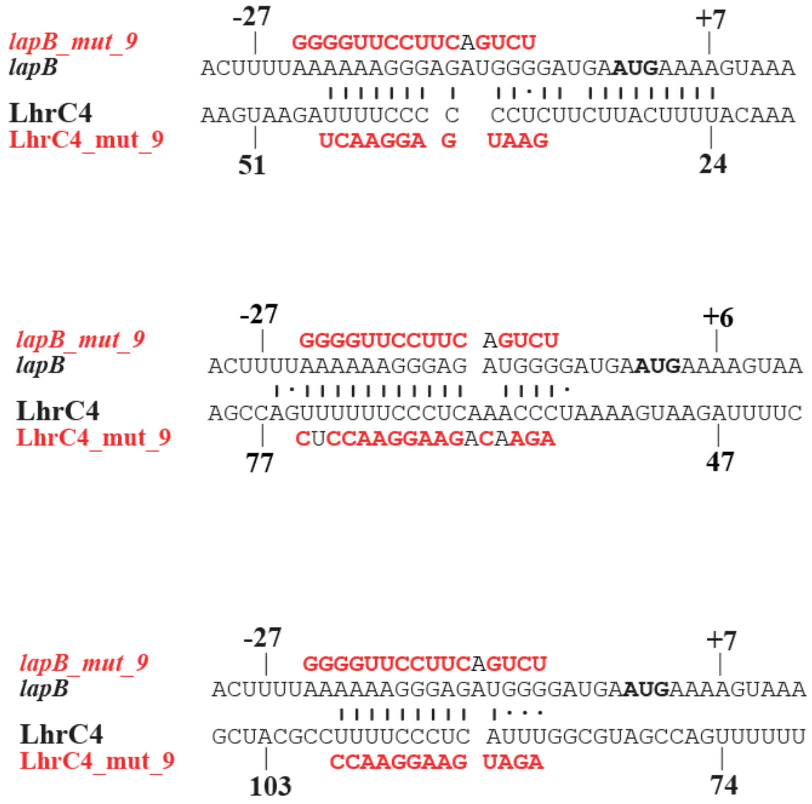

Figure S17. Schematic overview of substitutions in *lapB_mut9* and LhrC4_mut9. The three possible interactions of *lapB* with LhrC4 stem-loop A (upper panel), LhrC4 single stranded spacer region (middle panel) and LhrC4 terminator (lower panel), respectively, are shown. The mutations in *lapB_mut_9* and LhrC4_mut_9 are indicated in red.

Table S1. Primers used in this study.

Name	Sequence (5´→3´)	Further information
NB probes		
**lhrC_probe**	AATGAAAATGTTTTGCTTGTTGTTAGCTTAT	also for detection of LhrC4_mut
**lhrA_probe**	TTGCCATCATGTTCGGGC	
**5S_probe_Lmo**	GAGAAGCTTAACTACCGTGTTCGGGATGGGAACGG	
**5S_probe_Eco**	CTACGGCGTTTCACTTCTGAGTTCCCGTATGTAGCATC ACCTTC	
in-frame deletions		
**lhrC1-4_up_fw**	GGGGGAATTCCCAGTACTTAGTGGCGGTTCTCC	used in PCRI and III for ΔlhrC1-4 construction
**lhrC1-4_up_rev**	CCAAGAAAAGAAAGTTTCTGCTGTGC	used in PCRI for ΔlhrC1-4 construction; overlaps with lhrC1-4_down_fw
**lhrC1-4_down_fw**	CACAGCAGAAACTTTCTTTTCTTGGGTGCGCTAAAAT CACTAAATTCTGC	used in PCRII for ΔlhrC1-4 construction; overlaps with lhrC1-4_up_rev
**lhrC1-4_down_rev**	GGGGGGATCCCATATCAGCTCCCGCTAAGATAGCC	used in PCRII and III for ΔlhrC1-4 construction
**lhrC5_up_fw**	GGGGTCTAGATTGCCGAGCGACCATATCAG	used in PCRI and III for ΔlhrC5 construction
**lhrC5_up_rev**	ACATATAATAACTTTTCTAAAAAAGAATGTGC	used in PCRI for ΔlhrC5 construction; overlaps with lhrC5_down_fw
**lhrC5_down_fw**	GCACATTCTTTTTTAGAAAAGTTATTATATGTATGCGC AAAAATCACTTTTTCTGAG	used in PCRII for ΔlhrC5 construction; overlaps with lhrC5_up_rev
**lhrC5_down_rev**	CCCCGAATTCCCGGACGAATGAAAAATCAGC	used in PCRII and III for ΔlhrC5 construction
**lhrC1-4_flank_fw**	CGTACTTAAAAAGAACTACCCAGACG	flanking primer of lhrC1-4 locus for mutant verification
**lhrC1-4_flank_rev**	CGGTACATCGTATTTTGCAGC	flanking primer of lhrC1-4 locus for mutant verification
**lhrC5_flank_fw**	GCATTAATTTAAATACAGCATCTAGTGAAG	flanking primer of lhrC5 locus for mutant verification
**lhrC5_flank_rev**	GGTTATGCCGATTACGTTTATTATTC	flanking primer of lhrC5 locus for mutant verification
**hfq_A**	GGGGTCTAGAGAAGGTTTAGTGACAGAAGCG	used in PCRI and III for Δhfq construction
**hfq_B**	CATAATTTCCCTCTCCAATCTC	used in PCRI for Δhfq construction; overlaps with hfq_C
**hfq_C**	GAGATTGGAGAGGGAAATTATGCCTGATGCGGAATA AGCAC	used in PCRII for Δhfq construction; overlaps with hfq_B
**hfq_D**	CCCCGGATCCAGCCGAAATATTGCGCAC	used in PCRII and III for Δhfq construction
**hfq_flank_fw**	GGAGTATCAAGTGCATAATGTAC	flanking primer of hfq locus for mutant verification
**hfq_flank_rev**	GGTAAAAGATATTTATATTGCGC	flanking primer of hfq locus for mutant verification
transcriptional and translational fusions		
**lhrC1_p_fw**	GGGGGAATTCGAACGCTACTTAAGCACGC	transcriptional fusion of lhrC1 promoter in pTCV-lac

**lhrC1_p_rev**	GGGGGGATCCGCTTATGACTATATATTAACCAAGA	transcriptional fusion of lhrC1 promoter in pTCV-lac
**lhrC2_p_fw**	GGGGGGAATTCTCCCTTTTTCCACACTGGTTTTTTAATGCGAAAAATGAAAAGAAAAACCGAGAAATCTCATGAAACTTTAAC	transcriptional fusion of lhrC2 promoter in pTCV-lac
**lhrC2_p_rev**	GGGGGGATCCGCTTATGACTATATATTAACCAAGTTTTTGGTGGATACGCTGTGACTTTTGTTAAAGTTTCATGAGATTTCTCGG	transcriptional fusion of lhrC2 promoter in pTCV-lac
**lhrC3_p_fw**	GGGGGAATTCCCTTTTTCCACACTGGTTTTTTG	transcriptional fusion of lhrC3 promoter in pTCV-lac
**lhrC3_p_rev**	GGGGGATCCGCTTATAACTATATATTAACCAAGTTTTT	transcriptional fusion of lhrC3 promoter in pTCV-lac
**lhrC4_p_fw**	GGGGGGAATTCTCCCTTTTTCCACACTGGTTTTTTGATGCAAAAAATGAAAAGAAAAACCGAGAAATCTCATGAAACTTTAAC	transcriptional fusion of lhrC4 promoter in pTCV-lac
**lhrC4_p_rev**	GGGGGGATCCGCTTATGACTATATATTAACCAAGTTTTCGGTGGATACGCTGTGATATTTGTTAAAGTTTCATGAGATTTC	transcriptional fusion of lhrC4 promoter in pTCV-lac
**lhrC5_p_fw**	GGGGAATTCGAAAAATAGGATTGCAGAAAAGC	transcriptional fusion of lhrC5 promoter in pTCV-lac
**lhrC5_p_rev**	GGGGGATCCGCTTATACATATAATAACTTTTCTAAAA	transcriptional fusion of lhrC5 promoter in pTCV-lac
**lhrA_p_fw**	GGGGGAATTCCTTGCTTTTTTCAAGAACAATAGTAAAAT	transcriptional fusion of lhrA promoter in pTCV-lac
**lhrA_p_rev**	GGGGGGATCCTATAACTTATTTTACTATTGTTCTTGAAA	transcriptional fusion of lhrA promoter in pTCV-lac
**lapB_p_fw**	GGGGGAATTCTAAAAGACGCGGTTCGCGGGG	transcriptional fusion of lapB promoter in pTCV-lac
**lapB_p_rev**	GGGGGGATCCTTAAAAGTCCCCCAATTATTTTATCCC	transcriptional fusion of lapB promoter in pTCV-lac
**lmo1669_p_fw**	GGGGGAATTCGCGTTTTTATCTTTTTTAGGTGG	transcriptional fusion of lmo1669 promoter in pTCV-lac
**lmo1669_p_rev**	GGGGGGATCCATAAATAAACAAAAACTCACTTCC	transcriptional fusion of lmo1669 promoter in pTCV-lac
**pC_lapB_lacZ_fw**	GGGGGAATTCCTTGCTTTTTTCAAGAACAATAGTAAAATAAGTTATATCAAGTTTGTCATAGATAAAATAGGGGAATA	translational fusion of LhrA core promoter + lapB (-100 to +32 of translational start) in pCK-lac
**pC_lapB_lacZ_rev**	GGGGGGATCCGTCATCATGCCTATAAAAAATTTACTTTTCATTCAT	translational fusion of LhrA core promoter + lapB (-100 to +32 of translational start) in pCK-lac
**Vlac-1**	GTTGAATAACACTTATTCCTATC	flanking forward primer to check insertions in pTCV-lac and pCK-lac
**Vlac-2**	CTTCCACAGTAGTTACACCACC	flanking reverse primer to check insertions in pTCV-lac
**-40 primer**	GTTTTCCCAGTCACGACGTTGTAAAACGACGG	flanking reverse primer to check insertions in pCK-lac.
RT-qPCR		
**lmo0014_fw**	CGATACCGACCGGGATTAAGATATTCAAC	
**lmo0024_fw**	TGAAGAGCGAGAAAAGAAAGCATCAAAAG	
**lmo0024_rev**	CCCCGAAAATCGTATCACCAACACC	
**lmo0282_fw**	ACCTTCGTCGTTGCCGTCAATC	
**lmo0282_rev**	CAATGTTACCAAGTGGATCAATCACAAGC	

**lmo0286_fw**	GCCAGTCCCTGAAGACTTTACAAGTAGC	
**lmo0286_rev**	CCAAACTCCCCAAAACCACTACCATCC	
**lmo0412_fw**	GCTTAAACTAGCACTAACCCCTGCTTAC	
**lmo0412_rev**	CTACACGTTCGGCTTCAGCTTTTTC	
**lmo1041_fw**	GCTTATGCCAAACAAACGCTCGAAAAC	
**lmo1041_rev**	GGCTTCCACGTAGGATAAGACTTGAC	
**lmo1054_fw**	AAGGTACAGTTGCTACAGTTGGACAAG	
**lmo1054_rev**	TGCTTTTGGTGCTGCGCTTTC	
**lmo1493_fw**	TGGGCACGCATTCCATTCACAC	
**lmo1493_rev**	ATCGGCAATAATCATTTCCGCAAAAGTAG	
**lapB_fw**	TGATTCACCCACTTGAGACTCCAAAAC	
**lapB_rev**	TGCATGAATAGCATTTGCAGGGACTAC	
**lmo1872_fw**	ATATGATGCAGGTTGCGGGGAAGG	
**lmo1872_rev**	TCTAAGCCAACTGCTTTGACATTAACACC	
**lmo1993_fw**	CCACAAGCAAAATACCAAATCGAAGTACC	
**lmo1993_rev**	CGCCTAGAATCATTGCAGCGATTCC	
**lmo2353_fw**	GACTATATGTCCAAACACGAGCAGAGAAG	
**lmo2353_rev**	AGAGGAACAAATCGGAAGTCTCGAAAAC	
**actA_fw**	TCCCACCAATCCCAACAGAAGAAGAG	
**actA_rev**	TCGCTGTTTTCGTCATCTGTAAAATCACC	
**rpoB_fw**	CGTCGTCTTCGTTCTGTTGG	
**rpoB_rev**	GTTCACGAACCACACGTTCC	
**tpi_fw**	AACACGGCATGACACCAATC	
**tpi_rev**	CACGGATTTGACCACGTACC	
EMSA		
**T7_lapB_fw**	GGGGGAATTCTAATACGACTCACTATAGGGTATCAA GTTTGTCATAGATA	synthesis of lapB DNA with T7 promoter to be transcribed into RNA; also for structure probing; used in PCRsIII for mutant construction on PCRI and II products
**T7_lapB_rev**	GGGGGGATCCTGCTTCTGCTTTCATTCCA	synthesis of lapB DNA with T7 promoter to be transcribed into RNA; also for structure probing; used in PCRIII for mutant construction on PCRI and II products
**T7_lhrC4_fw**	GGGGGAATTCTAATACGACTCACTATAGGGATAAGC TAACAACAAACAAAACATTTTCATTCTTCTCCCCCCTT TTAGAATGAAAATCCC	synthesis of lhrC4 DNA with T7 promoter to be transcribed into RNA; also for structure probing; PCR with overlapping primers (no template)
**T7_lhrC4_rev**	GGGGGGATCCAAAAAAACCGATGCGGAAAAGGGAG TAAACCGCATCGGTCAAAAAAGGGAGTTTGGGATTT TCATTCTAAAAGGGG	synthesis of lhrC4 DNA with T7 promoter to be transcribed into RNA; also for structure probing; PCR with overlapping primers (no template)
**lapB_mut_1_rev**	AATTTACTTTTCATTCATCCCCATGAGGGAATTTTAAA AGTCCCCCAATTATTTTATC	use with T7_lapB_fw in PCRI; overlaps with lapB_mut_1_fw
**lapB_mut_1_fw**	GATAAAATAATTGGGGGACTTTTAAAATTCCCTCATG GGGATGAATGAAAAGTAAATT	use with T7_lapB_rev in PCRII; overlaps with lapB_mut_1_rev
**lhrC4_mut_2_fw**	GGGGGAATTCTAATACGACTCACTATAGGGATAAGC	use with overlapping

	TAACAACAAACAAAACATTTTCATTCTTCTCCCCCCTT TTAGAATGAAAATAGAACAG	lhrC4_mut_2_rev primer
**lhrC4_mut_2_rev**	GGGGGGATCCAAAAAAACCGATGCGGAAAAGGGAG TAAACCGCATCGGTCGGGGTTCCTTCTGTTCTATTTTC ATTCTAAAAG	use with overlapping lhrC4_mut_2_fw primer
**lhrC4_mut_3_fw**	GGGGGAATTCTAATACGACTCACTATAGGGATAAGC TAACAACAAACAAAACATTTTCATTCTTCTCCCCCCTT TTAGAATGAAAATCCCAAAC	use with overlapping lhrC4_mut_3_rev primer
**lhrC4_mut_3_rev**	GGGGGGATCCAAAAAAACCGATGCGGTTTTCCCTCT AGTCCGCATCGGTCAAAAAAGGGAGTTTGGGATTTT CATTCTAAAAG	use with overlapping lhrC4_mut_3_fw primer
**lhrC4_mut_4_fw**	GGGGGAATTCTAATACGACTCACTATAGGGATAAGC TAACAACAAACAAAACATTTTCATTCTAATGGCGGGA AAAAGAATGAAAATCCCAAAC	use with overlapping lhrC4_mut_4_rev primer
**lhrC4_mut_4_rev**	GGGGGGATCCAAAAAAACCGATGCGGAAAAGGGAG TAAACCGCATCGGTCAAAAAAGGGAGTTTGGGATTT TCATTCTTTTTC	use with overlapping lhrC4_mut_4_fw primer
**lhrC4_mut_5_fw**	GGGGGAATTCTAATACGACTCACTATAGGGATAAGC TAACAACAAACAAAACATTTTCATTCTTCTCCCCCCTT TTAGAATGAAAATAGAACAG	use with overlapping lhrC4_mut_5_rev primer
**lhrC4_mut_5_rev**	GGGGGGATCCAAAAAAACCGATGCGGTTTTCCCTCT AGTCCGCATCGGTCGGGGTTCCTTCTGTTCTATTTTCA TTCTAAAAG	use with overlapping lhrC4_mut_5_fw primer
**lhrC4_mut_6_fw**	GGGGGAATTCTAATACGACTCACTATAGGGATAAGC TAACAACAAACAAAACATTTTCATTCTAATGGCGGGA AAAAGAATGAAAATAGAACAG	use with overlapping lhrC4_mut_6_rev primer
**lhrC4_mut_6_rev**	GGGGGGATCCAAAAAAACCGATGCGGAAAAGGGAG TAAACCGCATCGGTCGGGGTTCCTTCTGTTCTATTTTC ATTCTTTTTC	use with overlapping lhrC4_mut_6_fw primer
**lhrC4_mut_7_fw**	GGGGGAATTCTAATACGACTCACTATAGGGATAAGC TAACAACAAACAAAACATTTTCATTCTAATGGCGGGA AAAAGAATGAAAATAGAACAG	use with overlapping lhrC4_mut_7_rev primer
**lhrC4_mut_7_rev**	GGGGGGATCCAAAAAAACCGATGCGGTTTTCCCTCT AGTCCGCATCGGTCGGGGTTCCTTCTGTTCTATTTTCA TTCTTTTTC	use with overlapping lhrC4_mut_7_fw primer
**lhrC4_mut_8_fw**	GGGGGAATTCTAATACGACTCACTATAGGGATAAGC TAACAACAAACAAAACATTTTCATTCTAATGGCGGGA AAAAGAATGAAAATCCCAAAC	use with overlapping lhrC4_mut_8_rev primer
**lhrC4_mut_8_rev**	GGGGGGATCCAAAAAAACCGATGCGGTTTTCCCTCT AGTCCGCATCGGTCAAAAAAGGGAGTTTGGGATTTT CATTCTTTTTC	use with overlapping lhrC4_mut_8_fw primer
**lhrC4_mut_stem_fw**	GGGGGAATTCTAATACGACTCACTATAGGGATAAGC TAACAACAAACAAAACTAAAAGTAAGATCTCCCCCCT TTTTCTTACTTTTACCCAAAC	use with overlapping lhrC4_mut_stem_rev primer
**lhrC4_mut_stem_rev**	GGGGGGATCCAAAAAAACCGATGCGGAAAAGGGAG TAAACCGCATCGGTCAAAAAAGGGAGTTTGGGTAAA AGTAAGAAAAAG	use with overlapping lhrC4_mut_stem_fw primer
**lapB_mut_stem_rev**	GAAAAGTAAGTTCCCCATCTCCCTTTTTTAA	use with T7_lapB_fw in PCRI; overlaps with lapB_mut_stem_fw
**lapB_mut_stem_fw**	GATGGGGAACTTACTTTTCTAAATTTTTTATAGGCAT GATG	use with T7_lapB_rev in PCRII; overlaps with lapB_mut_stem_rev
**lhrC4_mut_9_fw**	GGGGGAATTCTAATACGACTCACTATAGGGATAAGC TAACAACAAACAAAACATTTTCATTCTTGAATGAGGA ACTAGAATGAAAATAG	use with overlapping lhrC4_mut_9_rev primer
**lhrC4_mut_9_rev**	GGGGGGATCCAAAAAAACCGATGCGGGGTTCCTTCA	use with overlapping

Table S1, continued

	TCTCCGCATCGGTCGAGGTTCCTTCTGTTCTATTTTCATTCTAGTTCCTCA	lhrC4_mut_9_fw primer
**lapB_mut_9_rev**	CTTTTGGGGTTCCTTCAGTCTGATGAATGAAAAGTAAATTTTTTAT	use with T7_lapB_fw in PCRI; overlaps with lapB_mut_9_fw
**lapB_mut_9_fw**	CAGACTGAAGGAACCCCAAAAGTCCCCCAATTATTTTA	use with T7_lapB_rev in PCRII; overlaps with lapB_mut_9_rev
**lhrC4_mut_10_rev**	AAAAAAACCGATGCGGAAAACCGACTAAACCGCATCGGTCAAAAAAGGGAGTTTGGGATTTTCATTCTAAAAGGGG	use with overlapping T7_lhrC4_fw primer
**lapB_mut_10_rev**	TTTACTTTTCATTCATCCCCATGTCGGTTTTTTAAAAGTCCCCCAATTATTTTATC	use with T7_lapB_fw in PCRI; overlaps with lapB_mut_10_fw
**lapB_mut_10_fw**	GATAAAATAATTGGGGGACTTTTAAAAAACCGACATGGGGATGAATGAAAAGTAAA	use with T7_lapB_rev in PCRII; overlaps with lapB_mut_10_rev
GFP reporter experiments		
**lapB_pXG10_fw**	GGGGATGCATTCAAGTTTGTCATAGATAAAATAGGGG	synthesis of lapB fragment for insertion into pXG-10; also used in PCRIII for mutant construction on PCRI and II products
**lapB_pXG10_rev**	GGGCGCTAGCAAAAGTCATCATGCCTATAAAAAATTTACTTTTC	synthesis of lapB fragment for insertion into pXG-10; also used in PCRIII for mutant construction on PCRI and II products
**lhrC4_pNDM220_fw**	GCCTGACGTCGGCAAAAAGAGTGTTGACTTGTGAGCGGATAACAATGATACTTAGATTCATAAGCTAACAACAAACAAAACATTTTC	synthesis of lhrC4 for insertion into pNDM220; also used in PCRIII for mutant construction on PCRI and PCRII products
**lhrC4_pNDM220_rev**	CCCCGGATCCAAAAAAACCGATGCGGAAAAGGGAGTAAACCGCATCGGTCAAAAAAG	synthesis of lhrC4 for insertion into pNDM220; also used in PCRIII for mutant construction on PCRI and PCRII products
**mut_lapB_pXG10_rev**	CATTCATCCCCCCTCCTGAAATTTAAAAGTCCCCCAATTATTTTATCCC	use with lapB_pXG10_fw in PCRI; overlaps with mut_lapB_pXG10_fw
**mut_lapB_pXG10_fw**	CTTTTAAATTTCAGGAGGGGGGATGAATGAAAAGTAAATTTTTTATAG	use with lapB_pXG10_rev in PCRII; overlaps with mut_lapB_pXG10_rev
**mut_lhrC4 _pNDM220_rev**	CAAATTTCAGGACTGGGGATTTTCATTCTAAAAGGGGGGAGAAG	use with lhrC4_pNDM220_fw in PCRI; overlaps with mut_lhrC4 _pNDM220_fw
**mut_lhrC4 _pNDM220_fw**	GAATGAAAATCCCCAGTCCTGAAATTTGACCGATGCGGTTTACTCCC	use with lhrC4_pNDM220_rev in PCRII; overlaps with mut_lhrC4 _pNDM220_rev
**pZE-CAT**	TGGGATATATCAACGGTGGT	flanking primer to check insertions in pXG-10 (Urban & Vogel 2007)
**JVO-0155**	CCGTATGTAGCATCACCTTC	flanking primer to check insertions in pXG-10 (Urban & Vogel 2007)
**JMJ221**	TTGTCTCATGAGCGGATACA	flanking primer to check insertions in pNDM220
**JMJ207**	GGCCTCTTCGCTATTACGCG	flanking primer to check insertions in pNDM220

Table S2. Strains used in this study.

**Strain**	**Origin**
Listeria monocytogenes **serotype 1/2c LO28**	(6)
**LO28Δ*lisR***	(7)
**LO28Δ*cesR***	(8)
**LO28Δ*lhrC1-4***	this study
**LO28Δ*lhrC5***	this study
**LO28Δ*lhrC1-5***	this study
**LO28Δ*hfq***	this study
Escherichia coli **TOP10**	Invitrogen
Escherichia coli **DH5a**	Invitrogen

Table S3. RNA target hitlist. RNApredator (9) was used to search for direct RNA targets of LhrC1-5. The five top hits of each LhrC copy were combined in a single table. Given are accession numbers, gene names and searching ranks of the target RNAs.

accesion number	gene name	sequence bound by LhrC1 (rank)	sequence bound by LhrC2 (rank)	sequence bound by LhrC3 (rank)	sequence bound by LhrC4 (rank)	sequence bound by LhrC5 (rank)
lmo0014	*qoxB*				-19 -> -9 (5)	
lmo0024					-10 -> +8 (1)	
lmo0204	*actA*	-16 -> +7 (5)	-16 -> +7 (4)	-16 -> +7 (4)		
lmo0282					181 -> +189 (3)	
lmo0286		1065 -> +1075 (3)	1065 -> +1075 (2)	1065 -> +1075 (2)		
lmo0412						-22 -> -2 (3)
lmo1041					-16 -> +6 (2)	
lmo1054	*pdhC*	-85 -> -62 (1)				-83 -> -59 (1)
lmo1493		445 -> +458 (4)	445 -> +458 (3)	445 -> +458 (3)		
lmo1666	*lapB*	-19 -> -5 (2)	-19 -> -5 (1)	-19 -> -5 (1)	-20 -> -5 (4)	-18 -> -2 (4)
lmo1872			403 -> +416 (5)	403 -> +416 (5)		
lmo1993	*pdp*					-16 -> +9 (2)
lmo2353						1886 -> +1903 (5)

Table S4. RT-qPCR results of 13 putative targets for LhrC. RT-qPCR results of two biological replicates (each in technical duplicates) are presented for time points 30 min and 60 min for both non-stressed and stressed samples. Absolute ratios as well as log2 ratios of Δ*lhrC*1-5/wt and their technical standard deviations are given. In columns 3, 6, 9 and 12 biological parallels are averaged.

		1	2	3	4	5	6	7	8	9	10	11	12
**Accession number**		control 1st 30'	control 2nd 30'	control 30' average	control 1st 60'	control 2nd 60'	control 60' average	stress 1st 30'	stress 2nd 30'	stress 30' average	stress 1st 60'	stress 2nd 60'	stress 60' average
lmo0014	ratio mutant/wt	0,67	1,06	0,86	0,70	0,76	0,73	0,86	1,60	1,23	1,02	1,44	1,23
	standard deviation	0,05	0,15		0,05	0,12		0,05	0,14		0,07	0,17	
	log2 ratio mutant/wt	-0,58	0,09	-0,25	-0,52	-0,39	-0,46	-0,22	0,68	0,23	0,03	0,53	0,28
	standard deviation log2	0,02	0,01		0,02	0,03		0,01	0,06		0,00	0,06	
lmo0024	ratio mutant/wt	0,60	1,22	0,91	0,26	1,03	0,64	0,76	1,05	0,90	0,30	1,98	1,14
	standard deviation	0,05	0,18		0,02	0,14		0,07	0,10		0,01	0,21	
	log2 ratio mutant/wt	-0,73	0,29	-0,22	-1,93	0,04	-0,95	-0,39	0,07	-0,16	-1,73	0,98	-0,37
	standard deviation log2	0,02	0,04		0,01	0,00		0,02	0,01		0,01	0,10	
lmo0204	ratio mutant/wt	1,26	1,11	1,18	0,85	0,43	0,64	1,23	1,16	1,19	1,33	1,14	1,24
	standard deviation	0,08	0,20		0,06	0,06		0,10	0,16		0,09	0,19	
	log2 ratio mutant/wt	0,33	0,15	0,24	-0,24	-1,20	-0,72	0,30	0,21	0,26	0,41	0,19	0,30
	standard deviation log2	0,02	0,03		0,01	0,03		0,03	0,03		0,03	0,03	
lmo0282	ratio mutant/wt	1,07	1,01	1,04	0,88	0,83	0,86	0,19	1,18	0,69	1,33	1,70	1,51
	standard deviation	0,07	0,14		0,07	0,10		0,31	0,11		0,08	0,22	
	log2 ratio mutant/wt	0,10	0,01	0,05	-0,18	-0,26	-0,22	-2,37	0,24	-1,06	0,41	0,76	0,59
	standard deviation log2	0,01	0,00		0,01	0,02		0,14	0,02		0,02	0,10	
lmo0286	ratio mutant/wt	0,93	1,08	1,01	0,82	0,73	0,77	1,06	1,15	1,11	1,13	1,06	1,09
	standard deviation	0,08	0,15		0,04	0,08		0,05	0,12		0,14	0,11	
	log2 ratio mutant/wt	-0,10	0,12	0,01	-0,28	-0,46	-0,37	0,09	0,20	0,15	0,18	0,08	0,13
	standard deviation log2	0,01	0,02		0,01	0,03		0,00	0,02		0,02	0,01	
lmo0412	ratio mutant/wt	1,17	1,25	1,21	0,94	1,09	1,01	1,23	1,46	1,35	1,33	1,10	1,22
	standard deviation	0,11	0,19		0,07	0,12		0,08	0,13		0,07	0,11	
	log2 ratio mutant/wt	0,23	0,32	0,28	-0,09	0,13	0,02	0,30	0,55	0,43	0,41	0,14	0,28
	standard deviation log2	0,02	0,05		0,01	0,01		0,02	0,05		0,02	0,01	
lmo1041	ratio mutant/wt	1,02	1,15	1,09	0,67	1,08	0,87	1,27	1,89	1,58	1,97	2,32	2,14
	standard deviation	0,06	0,17			0,12		0,16	0,23		0,12	0,25	
	log2 ratio mutant/wt	0,03	0,21	0,12	-0,59	0,11	-0,24	0,35	0,92	0,63	0,98	1,21	1,10
	standard deviation log2	0,00	0,03			0,01		0,04	0,11		0,06	0,13	
		1	2	3	4	5	6	7	8	9	10	11	12
**Accession**		control	control	control	control	control	control	stress 1st	stress	stress 30'	stress 1st	stress	stress 60'

Table S4, continued

**number**		1st 30'	2nd 30'	30' average	1st 60'	2nd 60'	60' average	30'	2nd 30'	average	60'	2nd 60'	average
lmo1054	ratio mutant/wt	0,75	1,11	0,93	0,53	1,15	0,84	0,59	1,13	0,86	0,90	1,19	1,04
	standard deviation	0,05	0,17		0,05	0,12		0,04	0,11		0,06	0,13	
	log2 ratio mutant/wt	-0,42	0,15	-0,13	-0,93	0,20	-0,36	-0,76	0,18	-0,29	-0,16	0,25	0,04
	standard deviation log2	0,01	0,02		0,02	0,02		0,02	0,02		0,01	0,03	
lmo1493	ratio mutant/wt	1,02	1,02	1,02	0,89	0,76	0,82	1,21	1,20	1,20	1,50	1,27	1,38
	standard deviation	0,08	0,14		0,06	0,12		0,06	0,12		0,08	0,14	
	log2 ratio mutant/wt	0,03	0,02	0,03	-0,17	-0,39	-0,28	0,27	0,26	0,27	0,58	0,34	0,46
	standard deviation log2	0,00	0,00		0,01	0,03		0,01	0,03		0,03	0,04	
lmo1666	ratio mutant/wt	1,02	1,18	1,10	0,71	0,75	0,73	1,52	1,84	1,68	3,29	3,22	3,26
	standard deviation	0,06	0,16		0,05	0,09		0,15	0,21		0,31	0,35	
	log2 ratio mutant/wt	0,03	0,23	0,13	-0,49	-0,41	-0,45	0,61	0,88	0,74	1,72	1,69	1,70
	standard deviation log2	0,00	0,03		0,02	0,03		0,06	0,10		0,16	0,18	
lmo1872	ratio mutant/wt	1,07	1,11	1,09	0,95	0,83	0,89	0,91	0,94	0,93	1,53	1,15	1,34
	standard deviation	0,07	0,16		0,13	0,09		0,05	0,08		0,12	0,12	
	log2 ratio mutant/wt	0,10	0,15	0,12	-0,07	-0,27	-0,17	-0,14	-0,09	-0,11	0,61	0,20	0,41
	standard deviation log2	0,01	0,02		0,01	0,02		0,01	0,01		0,05	0,02	
lmo1993	ratio mutant/wt	0,99	1,24	1,12	0,55	0,88	0,71	0,98	1,26	1,12	1,21	2,22	1,72
	standard deviation	0,12	0,18		0,04	0,12		0,09	0,13		0,05	0,22	
	log2 ratio mutant/wt	-0,01	0,31	0,15	-0,87	-0,19	-0,53	-0,02	0,34	0,16	0,27	1,15	0,71
	standard deviation log2	0,00	0,04		0,02	0,02		0,00	0,04		0,01	0,12	
lmo2353	ratio mutant/wt	0,97	1,10	1,04	0,90	0,78	0,84	0,86	0,98	0,92	1,35	1,07	1,21
	standard deviation	0,10	0,15		0,06	0,11		0,06	0,12		0,19	0,11	
	log2 ratio mutant/wt	-0,04	0,13	0,05	-0,15	-0,35	-0,25	-0,22	-0,03	-0,12	0,44	0,09	0,27
	standard deviation log2	0,00	0,02		0,01	0,03		0,01	0,00		0,06	0,01	

Table S5. Measuring the promoter activity of *lapB* and *lmo1666*. The promoter regions of *lapB* and *lmo1666* were fused to *lacZ* in the transcriptional fusion vector pTCV-lac (4), resulting in plasmids pTCV-*lapB* and pTCV-*lmo1666*, respectively. The β-gal activity of LO28 wt and Δ*lhrC1-5* strains carrying pTCV-*lapB* and pTCV-*lmo1666* was determined after exposing the cells for 2 hours to 4 µg/ml cefuroxime. Unstressed cells were sampled as control. For comparison, the pC-*lapB-lacZ* construct was included in the same assay. The data correspond to the average of a single experiment performed in duplicate. The experiment was repeated twice, showing the same tendency.

	**pC-*lapB-lacZ***	**pTCV-*lapB***	**pTCV-*lmo1666***
**LO28 wt, control**	151	0	0
**Δ*lhrC1-5*, control**	150	0	0
**LO28 wt, stress**	134	0	0
**Δ*lhrC1-5*, stress**	307	0	0

Reference List

1. Lechat,P., Hummel,L., Rousseau,S. and Moszer,I. (2008) GenoList: an integrated environment for comparative analysis of microbial genomes. *Nucleic Acids Res.*, **36**, D469-D474.

2. Mraheil,M.A., Billion,A., Mohamed,W., Mukherjee,K., Kuenne,C., Pischimarov,J., Krawitz,C., Retey,J., Hartsch,T., Chakraborty,T. *et al.* (2011) The intracellular sRNA transcriptome of Listeria monocytogenes during growth in macrophages. *Nucleic Acids Res.*, **39**, 4235-4248.

3. Nielsen,J.S., Lei,L.K., Ebersbach,T., Olsen,A.S., Klitgaard,J.K., Valentin-Hansen,P. and Kallipolitis,B.H. (2010) Defining a role for Hfq in Gram-positive bacteria: evidence for Hfq-dependent antisense regulation in Listeria monocytogenes. *Nucleic Acids Res.*, **38**, 907-919.

4. Poyart,C. and Trieu-Cuot,P. (1997) A broad-host-range mobilizable shuttle vector for the construction of transcriptional fusions to beta-galactosidase in gram-positive bacteria. *FEMS Microbiol. Lett.*, **156**, 193-198.

5. Christiansen,J.K., Nielsen,J.S., Ebersbach,T., Valentin-Hansen,P., Sogaard-Andersen,L. and Kallipolitis,B.H. (2006) Identification of small Hfq-binding RNAs in Listeria monocytogenes. *RNA.*, **12**, 1383-1396.

6. Vazquez-Boland,J.A., Kocks,C., Dramsi,S., Ohayon,H., Geoffroy,C., Mengaud,J. and Cossart,P. (1992) Nucleotide sequence of the lecithinase operon of Listeria monocytogenes and possible role of lecithinase in cell-to-cell spread. *Infect. Immun.*, **60**, 219-230.

7. Kallipolitis,B.H., Ingmer,H., Gahan,C.G., Hill,C. and Sogaard-Andersen,L. (2003) CesRK, a two-component signal transduction system in Listeria monocytogenes, responds to the presence of cell wall-acting antibiotics and affects beta-lactam resistance. *Antimicrob. Agents Chemother.*, **47**, 3421-3429.

8. Gottschalk,S., Bygebjerg-Hove,I., Bonde,M., Nielsen,P.K., Nguyen,T.H., Gravesen,A. and Kallipolitis,B.H. (2008) The two-component system CesRK controls the transcriptional induction of cell envelope-related genes in Listeria monocytogenes in response to cell wall-acting antibiotics. *J. Bacteriol.*, **190**, 4772-4776.

9. Eggenhofer,F., Tafer,H., Stadler,P.F. and Hofacker,I.L. (2011) RNApredator: fast accessibility-based prediction of sRNA targets. *Nucleic Acids Res.*, **39**, W149-W154.
